# Spatially fractionated GRID radiation potentiates immune-mediated tumor control

**DOI:** 10.1186/s13014-024-02514-6

**Published:** 2024-09-13

**Authors:** Rebecca A. Bekker, Nina Obertopp, Gage Redler, José Penagaricano, Jimmy J. Caudell, Kosj Yamoah, Shari Pilon-Thomas, Eduardo G. Moros, Heiko Enderling

**Affiliations:** 1 Department of Integrated Mathematical Oncology, H. Lee Moffitt Cancer Center & Research Institute, Tampa, FL 33612 USA; 2 Department of Immunology, H. Lee Moffitt Cancer Center & Research Institute, Tampa, FL 33612 USA; 3 Department of Radiation Oncology, H. Lee Moffitt Cancer Center & Research Institute, Tampa, FL 33612 USA; 4 Cancer Biology Ph.D. Program, University of South Florida, Tampa, FL 33612 USA; 5 Department of Radiation Oncology, The University of Texas MD Anderson Cancer Center, Houston, TX 77030 USA; 6 Institute for Data Science in Oncology, The University of Texas MD Anderson Cancer Center, Houston, TX 77030 USA

**Keywords:** Tumor immune interactions, Spatially fractionated radiotherapy, Mathematical model, Personalized oncology

## Abstract

**Background:**

Tumor-immune interactions shape a developing tumor and its tumor immune microenvironment (TIME) resulting in either well-infiltrated, immunologically inflamed tumor beds, or immune deserts with low levels of infiltration. The pre-treatment immune make-up of the TIME is associated with treatment outcome; immunologically inflamed tumors generally exhibit better responses to radio- and immunotherapy than non-inflamed tumors. However, radiotherapy is known to induce opposing immunological consequences, resulting in both immunostimulatory and inhibitory responses. In fact, it is thought that the radiation-induced tumoricidal immune response is curtailed by subsequent applications of radiation. It is thus conceivable that spatially fractionated radiotherapy (SFRT), administered through GRID blocks (SFRT-GRID) or lattice radiotherapy to create areas of low or high dose exposure, may create protective reservoirs of the tumor immune microenvironment, thereby preserving anti-tumor immune responses that are pivotal for radiation success.

**Methods:**

We have developed an agent-based model (ABM) of tumor-immune interactions to investigate the immunological consequences and clinical outcomes after $2\,\text{Gy} \times 35$ whole tumor radiation therapy (WTRT) and SFRT-GRID. The ABM is conceptually calibrated such that untreated tumors escape immune surveillance and grow to clinical detection. Individual ABM simulations are initialized from four distinct multiplex immunohistochemistry (mIHC) slides, and immune related parameter rates are generated using Latin Hypercube Sampling.

**Results:**

In silico simulations suggest that radiation-induced cancer cell death alone is insufficient to clear a tumor with WTRT. However, explicit consideration of radiation-induced anti-tumor immunity synergizes with radiation cytotoxicity to eradicate tumors. Similarly, SFRT-GRID is successful with radiation-induced anti-tumor immunity, and, for some pre-treatment TIME compositions and modeling parameters, SFRT-GRID might be superior to WTRT in providing tumor control.

**Conclusion:**

This study demonstrates the pivotal role of the radiation-induced anti-tumor immunity. Prolonged fractionated treatment schedules may counteract early immune recruitment, which may be protected by SFRT-facilitated immune reservoirs. Different biological responses and treatment outcomes are observed based on pre-treatment TIME composition and model parameters. A rigorous analysis and model calibration for different tumor types and immune infiltration states is required before any conclusions can be drawn for clinical translation.

**Supplementary Information:**

The online version contains supplementary material available at 10.1186/s13014-024-02514-6.

## Background

Head and neck cancer (HNC) is one of the most prevalent cancer types globally, with cases rising in incidence [1]. Head and neck squamous cell carcinomas (HNSCC) represent 90% of HNC, for which treatment (Tx) options include radiotherapy (RT), surgical resection, and chemotherapy. Traditionally, RT was thought to be only genotoxic: inducing irreparable DNA damage leading to cell death. However, an extensive body of work now exists describing the interplay between radiotherapy and the immune system. RT-induced DNA damage leads to cell death and the release of tumor antigens and damage associated molecular patterns (DAMPs). These so-called danger signals initiate and potentiate innate and adaptive immune responses. For instance, calreticulin binds with CD91 on macrophages to promote phagocytosis, while HMGB1 binds with certain toll-like receptors (TLR2, TLR3, TLR4 and TLR9) to promote dendritic cell activation and the expression of pro-inflammatory cytokines such as NF-κB, TNF-α, and IL-6. Activated dendritic cells take up and process the released tumor antigens, before migrating to the lymph nodes to prime and activate tumor specific T cells, including cytotoxic CD8+ T cells [2–4]. Paradoxically, radiotherapy can also provoke immunosuppressive effects, by killing radiosensitive immune cells and inducing the downregulation of immune cell activation pathways while promoting the recruitment of immunosuppressive cell-types such as FOXP3 + CD4+ regulatory T cells and upregulating the PD-1–PD-L1 axis [5, 6]. The presence of CD8+ T cells has been positively correlated with response in multiple solid cancers, including HNC [7–9]. However, research into the relationships between other immune cells within the tumor-immune microenvironment (TIME) is not conclusive [10]. Thus, it is conceivable that patient response to RT may benefit from protecting the existing and/or induced anti-tumor immune subpopulations within the TIME.

One potential method of eradicating immunosuppressive populations whilst preserving and promoting immune effector cells is spatially fractionated radiotherapy (SFRT), developed in the early twentieth century to deliver high dose levels to deep tumors while minimizing the skin toxicity generally associated with the kilovoltage energy X-ray equipment of that time [11–13]. Since the 1950’s, SFRT has been used primarily in the palliative setting [14], or for debulking large tumors prior to conventional radiation, concomitant chemoradiotherapy, or surgery [15–18]. Current clinical use of SFRT focuses on single ablative doses ($> 15 \,\text{Gy}$) prior to conventional whole tumor/whole field RT (WTRT) [19, 20], which may obliterate the immune-activating features of SFRT. Recent insights into both pro- and anti-tumor immunological consequences of radiation warrant a novel look at SFRT: radiation-shielded areas may create immune reservoirs that are additionally promoted by the release of immune-stimulatory cytokines in adjacent unshielded sites. In fact, murine models of SFRT demonstrate increased systemic anti-tumor immunity [21], and indicate that tumor response correlates to radiation dosimetry parameters and the specific SFRT geometry (number of unshielded areas and distance between them) [22]. Other preclinical studies demonstrate that RT applied to only half of a tumor delays tumor growth longer than expected in immunocompetent but not immunodeficient mice, indicating a critical role of the immune system. This study further demonstrated CD8^+^ T-cell migration between the tumor periphery, the irradiated area, and the unirradiated tumor area [23]. To improve the clinical application of SFRT a deeper understanding of the immunological consequences of SFRT as a function of dose, dose fractionation, and SFRT geometry is required.

To exhaustively evaluate every such combination in even the pre-clinical setting is, however, infeasible [24]. Integrating mathematical modeling with experimental approaches and clinical data may circumvent these restrictions [25–34], while further elucidating relationships between the dose, dose fractionation, SFRT geometry and tumor response. Mathematical oncology models have been used in various contexts, from generating hypotheses of novel tumor biology to predicting treatment response [35]. The ultimate goal of modeling—hypothesis generation or prediction – generally informs methodological approaches. Models used for the latter should be rigorously calibrated with existing data, prior to being validated on unseen data, and the predictive power evaluated. Only then can the model be used to predict alternative treatments [36]. In contrast, models used for hypothesis generation only need to qualitatively recapitulate the system or phenomenon being studied.

Furthermore, the scientific question and available data inform the mathematical approach: deterministic modeling methods such as ordinary differential equations are ideal when working with temporal data on a population scale. However, stochastic modeling methods such as agent-based models (ABMs) are more suited to highly granular data such as tumor-immune cell interactions. ABMs are considered “bottom-up”, incorporating biological mechanism via individual autonomous agents which follow predetermined rules based on the underlying biology. These models facilitate investigations into how perturbations of the system or changes to the agent rules impact the emergent behavior of the model.

In this work, we developed such an ABM to explore relationships between SFRT GRID-block geometry, pre-treatment TIME states, and tumor response. Specifically, we investigated whether SFRT-GRID exhibits improved, or comparable efficacy compared to WTRT when administered to specific pre-Tx TIMEs**.**

## Methods

**Fig. 1:**
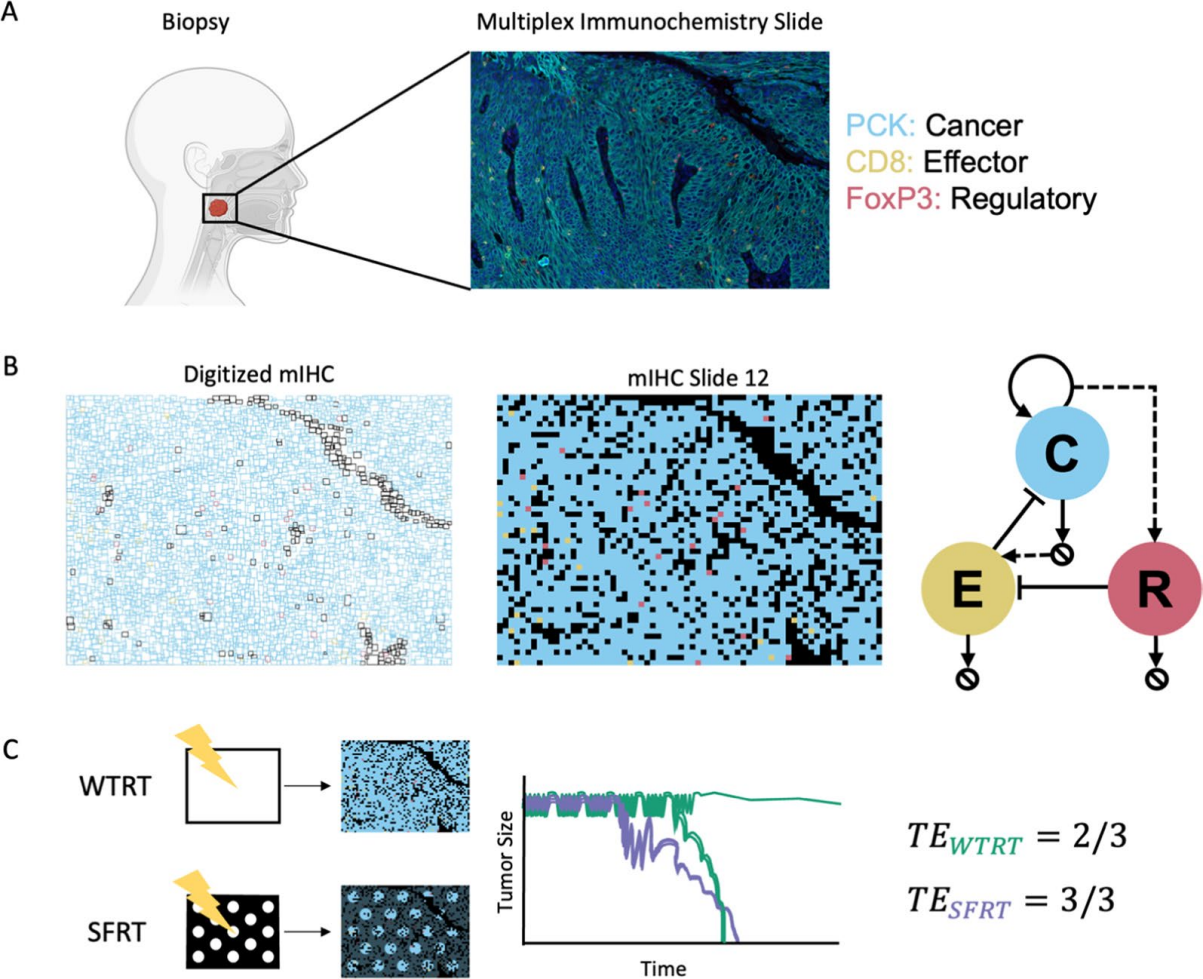
From patient biopsy to agent-based model.** A** Fluorescent Multiplex Immunohistochemistry (mIHC) is performed on biopsies of head and neck cancer to identify and quantify cellular populations within the tumor-immune microenvironment (TIME). **B** Digitized mIHC slides are used to generate in silico tumors, consisting of cancer cells (C), effector immune cells (E), and regulatory immune cells (R). See Methods for in-depth discussion of agent rules. **C** We treat in silico tumors from **B** with either WTRT or SFRT, and asses the treatment efficacy for each therapy

Herein we present a 2D on-lattice ABM of three populations, cancer cell agents (C), anti-tumor effector cell agents (E) and pro-tumor regulatory cell agents (R). This ABM is initialized from digitized fluorescent multiplex immunohistochemistry (mIHC) slides obtained from biopsies of primary HNC tumors. In silico treatment simulations using WTRT or two SFRT geometries are analyzed using MATLAB 2022, and the mechanisms of response and outcome are investigated (Fig. 1).

### Patient data

Patients with oropharyngeal cancer were identified via two IRB approved studies: Total Cancer Care (MCC#14690) with a diagnosis of head and neck cancer and obtained written consent, and Using Radiotherapy to Perturb the Tumor-Immune Ecosystem for Immune-Modulated Tumor Control (MCC#19233). After Institutional IRB approval was obtained, we selected four patients with different tumor immune ecosystem compositions for this study.

### Fluorescent multiplex immunohistochemistry (IHC) panel procedure

Formalin-fixed and paraffin-embedded (FFPE) tissue samples were immunostained using the AKOAYA Biosciences OPAL TM 7-Color Automation IHC kit (Waltham, MA) on the BOND RX autostainer (Leica Biosystems, Vista, CA). The OPAL 7-color kit uses tyramide signal amplification (TSA)-conjugated to individual fluorophores to detect various targets within the multiplex assay. Sections were baked at 65 °C for one hour then transferred to the BOND RX (Leica Biosystems). All subsequent steps (ex., deparaffinization, antigen retrieval) were performed using an automated OPAL IHC procedure (AKOYA). OPAL staining of each antigen occurred as follows: heat induced epitope retrieval (HIER) was achieved with Citrate pH 6.0 buffer for 20 min at 95 °C before the slides were blocked with AKOYA blocking buffer for 10 min. Then slides were incubated with primary antibody, CD68 (CST, D4BAC, 1:300, dye 520) at RT for 60 min followed by OPAL HRP polymer and one of the OPAL fluorophores during the final TSA step. Individual antibody complexes are stripped after each round of antigen detection. This was repeated five more times using the following antibodies; CD8 (DAKO, C8/144B, HIER-EDTA pH 9.0, 1:100, dye540), CD4 (CM, EP204, HIER- EDTA pH 9.0, 1:100, dye570), CD3 (Thermofisher, SP7, HIER-EDTA pH 9.0, 1:500, dye 570), FOXP3 (ABCAM, 236A/E7, HIER- EDTA pH 9.0, 1:500, dye650), and PCK (DAKO, AE1/AE3, HIER- Citrate pH 6.0, 1:200, dye690). After the final stripping step, DAPI counterstain is applied to the multiplexed slide and is removed from BOND RX for coverslipping with ProLong Diamond Antifade Mountant (ThermoFisher Scientific). All slides were imaged with the Vectra®3 Automated Quantitative Pathology Imaging System. See Table S1 for an overview of the stains used in the multiplex immunohistochemistry panel.

### Quantitative image analysis

Multi-layer TIFF images are exported from InForm (AKOYA) and loaded into HALO (Indica Labs, New Mexico) for quantitative image analysis. A classifier is trained to identify areas of tumor, stroma or non-tissue regions. Pan-cytokeratin is used to train tumor regions because it is a masking marker for tumor cells. The classifier is created and tested on various images in the image set. The tissue is segmented into individual cells using the DAPI marker which stains cell nuclei. For each marker, a positivity threshold within the nucleus or cytoplasm are determined per marker based on published staining patterns and intensity for that specific antibody. After setting a positive fluorescent threshold for each staining marker, the entire image set is analyzed with the created algorithm. The generated data includes positive cell counts for each fluorescent marker in cytoplasm or nucleus, and percent of cells positive for the marker. Along with the summary output, a per-cell analysis can be exported to provide the marker status, classification, and fluorescent intensities of every individual cell within an image.

### Agent-based model of tumor-immune interactions

The ABM was implemented in Java 1.8, using the Java library HAL (Hybrid Automata Library) [37], and is initialized from multiplex immunohistochemistry slides of head and neck cancer as detailed in the following section. The ABM domain represents an area of approximately $1.38 \;{\text{mm}}^{2}$, and consists of $68 \times 51$ nodes, with a domain constant of $20 \;\mu {\text{m}}$ × $20 \,\upmu\text{m}$, corresponding to the assumption of an average cell diameter of $20\,\upmu\text{m}$ (see following subsection and [38, 39]). The ABM timestep is $\Delta t=1\,\text{h}$. Each agent is autonomous, with their behavior being determined by cell-type specific rules based on the underlying biology. The specific rules are discussed in-depth in the following subsections. Due to the stochasticity of agent-based modeling, 50 independent replicates are run.

### ABM Initialization

HALO post processed files containing cellular locations ($xMin, yMin, xMax$ and $yMax$ coordinates) and types (see above) were imported into MATLAB. Next, we compute the cell center coordinates:

$$\left({x}_{computed},{y}_{computed}\right)= \left(\frac{xMin+xMax}{2},\frac{yMin+yMax}{2}\right)$$

which are mapped onto a 2D lattice by using the formula:

$${\left(x,y\right)}_{mapped}=floor\left(\frac{{x}_{computed}}{\kappa },\frac{{y}_{computed}}{\kappa }\right),$$

where $\kappa$ is a conversion factor. We select $\kappa =20,$ which corresponds to the assumption of an average cellular diameter of $20\,\upmu\text{m}$. The range $\left[\text{10,100}\right]$ for $\kappa$ was investigated and an inverse relationship between $\kappa$ and the fraction of retained cells within each in silico tumor can be observed (Fig. S1).

The mapped cell centers for each discretized mIHC slide are used to create the corresponding in silico tumor which is used as the initial condition of the ABM. Certain properties of individual agents are initialized by sampling from the corresponding distributions. For example, cancer cell agents keep track of their own cell cycle length, as well as their temporal position in their cell cycle. When a cancer cell agent is created during the initialization of the ABM, we assign the cell cycle length ($di{v}_{length}$) by sampling from a truncated normal distribution centered around 24 h. Then, we sample from the range $\left[0,\frac{di{v}_{length}}{2}\right]$ to assign the cell’s position in its cell cycle. The properties for which this is done can be found in Table S2–S4.

### Cellular processes and interactions

#### Cancer cell agent processes

Cancer cells can undergo apoptosis, proliferate, or migrate according to specific probabilities, and certain environmental restrictions (Fig. S2). Cancer cells progress through their cell cycle each timestep in which there is a vacant domain node in their Moore neighborhood. Once a cancer cell has reached the end of their cell cycle, mitosis may occur, and the daughter cell is placed in a randomly chosen vacant node within the Moore neighborhood. Cancer cells that are surrounded by neighbors are considered quiescent during that timestep, due to space inhibition. Cancer cells have a probability of apoptosis of ${p}_{a}= 3 \times {10}^{-3}\, \text{per timestep},$ which translates to a lifespan of approximately 14 days [40]. Cells that undergo apoptosis are removed from the simulation and the domain node becomes vacant immediately. The probability of migration of cancer cells is assumed to be ${p}_{m}=0.9\, \text{per timestep}$ (i.e., 1/h) and we assume a migration speed of $2.3 \,\upmu\text{m}/\text{min}$, this translates to a migration potential of 6 domain nodes per timestep. Thus, the migration of cancer cells is implemented as an iterative process, with cells moving up to 6 times per timestep [41, 42].

### Immune recruitment parameter rates

The ABM contains four immune recruitment related parameters. We estimate plausible values for these by generating 15 parameter sets using Latin Hypercube Sampling. Each set consists of a rate of (i) recruitment of effector-immune cells due to cancer cell apoptosis (${\zeta }_{apoptosis}$), (ii) recruitment of effector-immune cells due to effector-cell induced cancer cell death (${\zeta }_{effector}$), (iii) the probability of placing an effector cell (recruited in (ii)) near the location of cancer cell death $(\mu )$, and (iv) recruitment of regulatory cells due to cancer cell proliferation $({\zeta }_{regulatory})$ (see Table S5).

### Immune cell dynamics

#### Recruitment of Effector agents

Effector cell are recruited by the death of cancer cell agents, and so we record the number of apoptotic cancer cell deaths ${C}_{apoptosis}({t}_{i})$, and the number of cancer cell deaths due to interaction with effector immune cells ${C}_{effector}({t}_{i})$ that occur in timestep ${t}_{i}$. Then, the number of effector cells recruited in the next timestep${t}_{i+1}$, is:

$$\begin{aligned} & E_{apoptosis} \left( {t_{i + 1} } \right) = \zeta_{apoptosis} *C_{apoptosis} \left( {t_{i} } \right), \\ & E_{effector } \left( {t_{i + 1} } \right) = \zeta_{effector} *C_{effector} \left( {t_{i} } \right), \\ \end{aligned}$$

where ${\zeta }_{apoptosis}$ and ${\zeta }_{effector}$ are the rates at which the different types of cancer cell death recruit effector immune cells. Effector cell agents that are recruited due to apoptosis of cancer cells are placed randomly, following the assumption of a uniform distribution of blood vessels in the ABM domain. However, effector cell agents that are recruited due to the killing of cancer cells by effector immune cells are placed near locations of such death with probability $\mu$ (in an empty lattice node within a radius of $10$ lattice points of the cell-death location). This is in line with the so-called “post-code” hypothesis, which explains the multi-step, tissue-selective homing of T cells [43, 44].

#### Recruitment of regulatory agents

Regulatory cells are recruited by cancer cell proliferation events, and so for each timestep ${t}_{i}$ we record the number of proliferation events ${C}_{mitosis}({t}_{i})$. Then, the number of regulatory cells recruited in the next timestep ${t}_{i+1}$ is:

$$R\left({t}_{i+1}\right)={\zeta }_{regulatory}*{C}_{mitosis}\left({t}_{i}\right),$$

where ${\zeta }_{regulatory}$ is the rate of recruitment. Regulatory cells are placed in empty lattice nodes, randomly throughout the ABM domain.

### Migration

Immune cells migrate into, and infiltrate tumors by following specific biochemical gradients [44, 45], before interacting with their target cells (effector immune cells target cancer cells and regulatory immune cells target effector immune cells). We assume immune cell migration to be either entirely random or a combination of random- and directed motion towards their respective target cells. Similar methods of migration can be found elsewhere [46–48]. Below is the migration process for any immune cell $p$.

1. Find $q$ the nearest target cell(s) to $p$ within a radius of $r=50$ domain nodes.
  - If no target cells are found, immune cell $p$ undergoes random motion, moving into an open node within its Moore neighborhood.
  - If target cells are found:
    i. Calculate the normalized vector $\overline{u}$ from the location of cell $p$ to the location of cell* q*:
      $$\overline{u} = {{\frac{\overrightarrow{pq}}{\left\|{pq}\right\|}}}.$$
    ii. Calculate a random unit vector $\overline{v }$.
    iii. Calculate direction vector $\overline{w }$: $\overline{w }={\eta }_{rnd}\overline{v }+{\eta }_{directed}\overline{u }$, where ${\eta }_{rnd}$ and ${\eta }_{directed}$ are weights for random migration and directed migration respectively. (Note: ${\eta }_{rnd}=1-{\eta }_{directed}$ so that if ${\eta }_{rnd}=0$ immune cells undergo purely directed motion, while if ${\eta }_{directed}=0$ immune cells exhibit Brownian motion).
    iv. Find all the vacant lattice node(s) $x$ within the Moore neighborhood of $p$ that minimize the angle $\theta$ between the vectors $\overrightarrow{px}$ and $\overline{w }$.
    v. Randomly select one such node for $p$ to move into.

Immune cells are assumed to have a migration speed of $5 \,\upmu\text{m} / \text{min}$ [49–51], which translates to $15$ lattice nodes per timestep, and we fix ${\eta }_{directed}=0.19$. Thus, the above process is repeated multiple times per immune cell per timestep.

### Cell–cell interactions

Following attempted or successful migration by an immune cell $p$, it can interact with other cells. Recent studies have shown support for the “multiple hit” hypothesis, in which target cells require multiple contacts from cytotoxic lymphocytes before death [52]. It has also been shown that cells can recover from sublethal damage caused by interactions with cytotoxic lymphocytes, with those receiving two or more “hits” displaying accelerated apoptosis induction [53]. Thus, we assume that target cells require three “hits” with cells of the appropriate immune sub-type, and that the damage from these hits is repaired within a time step. That is, interaction with effector cells or regulatory cells will lead to the death of a cancer cell or effector cell respectively if three such interactions occur within a single timestep. However, we assume that each immune cell can only deliver a single hit during one timestep, with a maximum of 10 hits before becoming exhausted and being removed from the simulation (Fig. S2).

### Effects of radiation on cells

We calculate the probability that a cell of type $i$ survives a radiation dose of $d \,\text{Gy}$ using the linear quadratic equation (54, 55):

$$S{F}_{i}\left(d\right)=\text{exp}\left(-{\alpha }_{i}\frac{d}{\xi }-{\beta }_{i} {\left(\frac{d}{\xi }\right)}^{2}\right),$$

where ${\alpha }_{i} \left(\text{G}\text{y}^{-1}\right) \text{and }{\beta }_{i} (\text{G}\text{y}^{-2})$ are cell type specific radiosensitivity parameters [54]. For the purposes of this study we use $S{F}_{C}\left(2\,\text{Gy}\right)= 0.49, S{F}_{E}\left(2\,\text{Gy}\right)= 0.60$ and $S{F}_{R}\left(2\,\text{Gy}\right)= 0.77$ in line with those used previously [56]. As discussed elsewhere, proliferating cells are more sensitive to radiation than quiescent cells which we model by scaling the dose by $\xi =1$ if the cell is actively cycling, and $\xi =3$ if the cell is not [57, 58].

Radiation induces DNA damage [59, 60], the attempted repair of which may lead to temporary cell cycle arrest and successful repair or delayed cell death after failed repair. Studies show that these delays are dose dependent, and we assume a cell cycle lengthening of $2\,\text{h}/\text{Gy}$ [61], and an $8\,\text{h}$ delay between the administration of radiation and cell death in the case of irreparable DNA damage [62]. Thus, cancer cell killing via radiation is *not* immediate in this model.

### Radiation-induced effector immune cell recruitment

As previously mentioned, radiation-induced cancer cell death can be immunogenic, the level of which is determined by a range of factors (levels of tumor antigenicity, release of DAMPs, maturation of dendritic cells and other antigen presenting cells etc.) [6]. We collapse this biology into a single rate and assume that radiation-induced cancer cell death recruits effector immune cells at a rate of ${\zeta }_{Tx}$ immune effector cells per Tx-induced cancer cell death in the preceding timestep. Thus, radiation-induced cancer cell death recruits effector-immune cells at a rate

$${E}_{Tx}\left({t}_{i+1}\right)= {\zeta }_{Tx}*{C}_{Tx}\left({t}_{i}\right) .$$

For the purposes of this study, we assume radiation-induced cancer cell death is either non-, low- or highly immunogenic: ${\zeta }_{Tx}\in (0;0.01;0.1)$. That is, if ${\zeta }_{Tx}=0.1$, ten Tx-induced cancer cell deaths are required in timestep ${t}_{i}$ to recruit one effector immune cell in timestep ${t}_{i+1}$. Effector cells recruited in this manner are seeded randomly in the domain.

### Treatment with whole tumor radiotherapy

When treating with whole tumor radiotherapy (WTRT), a uniform dose of $2\,\text{Gy}$ radiation per weekday fraction is administered throughout the ABM domain, for a total dose of $70\,\text{Gy}$.

### Generation of in silico SFRT-GRID geometries

**Fig. 2:**
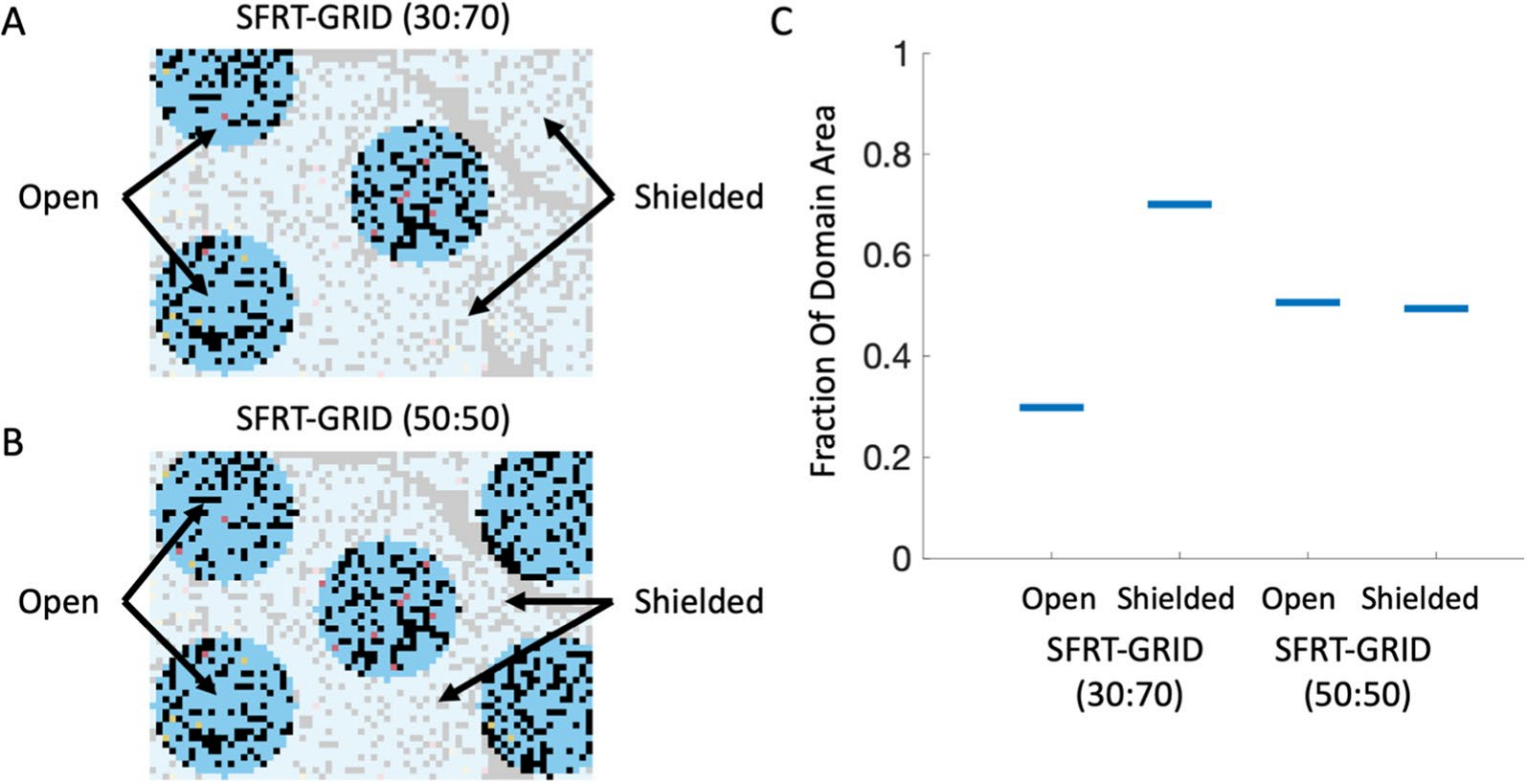
Characterization of the architectures of the in silico GRID blocks.** A**, **B** In silico SFRT-GRID blocks overlayed on mIHC slide 12, showing “peak” regions of the domain that appear under the openings of the GRID block and receive the full administered dose vs the areas in the shielded / “valley” regions which receive 15% of the administered dose. **C** Open to Shielded regions for both SFRT-GRID blocks. Approximately 30% of the ABM domain is in the “peak” areas, with approximately 70% being in the “valley” regions for the first geometry, while the second is an approximately even split of peak to valley areas

Commercially available SFRT collimator geometries have openings with diameters between 0.6–1 cm and center-to-center spacings of 1.15–1.4 cm, and generally result in approximately 50% of the tissue being shielded from the RT beam. These dimensions are not suited for the spatial scale of our agent-based model, and we scale the SFRT-GRID geometries appropriately. Two SFRT GRID block geometries are investigated here. The first has openings with a diameter of 11 lattice nodes and a center-to-center distance of 30 lattice nodes ($200\,\upmu\text{m}$ and $600 \,\upmu\text{m}$ respectively), and the second block has openings with a diameter of 11 lattice nodes and a center-to-center distance of 35 lattice nodes ($200\,\upmu\text{m}$ and $700 \,\upmu\text{m}$ respectively) (Fig. 2A,B). The first SFRT geometry results in shielding of approximately 70% of tissue, with the other 30% receiving the peak dose. The second SFRT geometry is in line with clinical practice and results in sparing of approximately 50% of the tissue, with the remaining 50% receiving the peak dose (Fig. 2C). For the rest of the manuscript, we refer to treatment using each geometry as SFRT-GRID (30:70) and SFRT-GRID (50:50) respectively. In line with published peak to valley dose ratios (PVDR) used in pre-clinical studies [22, 63, 64], we assume a PVDR of 15. That is, tissues within the dose valleys receive 15% of the peak dose per treatment fraction. For an administered radiation dose of $2\,\text{Gy}$, the average dose delivered to the entire domain for SFRT-GRID(30:70) and SFRT-GRID(50:50) is $0.81\,\text{Gy}$ and $1.15\,\text{Gy}$, respectively.

### Assessment of treatment success

The success of a treatment administered for a specific duration with the assumption of a specific level of immunogenicity of radiation-induced cell death ${\zeta }_{Tx}$, is described via the *probability of tumor eradication* of fifty independent simulations:

$$TE_{Tx} = { }\frac{{number\;{ }of\;{ }simulations{ }\;that{ }\;have{ }\;C = 0{ }\;at{ }\;end{ }\;of\;{ }simulation{ }}}{50}.$$

Each ABM initial condition was either left untreated and followed for 15 weeks or $2\,\text{Gy}$ weekday fractions were administered for 7 weeks and followed post-Tx for 8 weeks (for a possible total simulated time of 15 weeks). Simulations stopped at end the of week 15, or when the cancer population was entirely eradicated, whichever occurred first.

### Determining the primary mechanism of cancer cell death

For each timestep, the ABM output includes the number of cancer cells that undergo a specific type of cell death: effector cell cytotoxicity, direct radiation cell-kill, or apoptosis. These data are used to determine the primary mechanism of cancer cell death during the 7-day and 1-day periods immediately prior to and including the timestep in which tumor eradication occurs. Specifically, we calculate the total number of cancer cells which have undergone each type of death within the specific period.

## Results

### Agent-based model simulations without treatment exhibit distinct outcomes as a function of model parameters

**Fig. 3:**
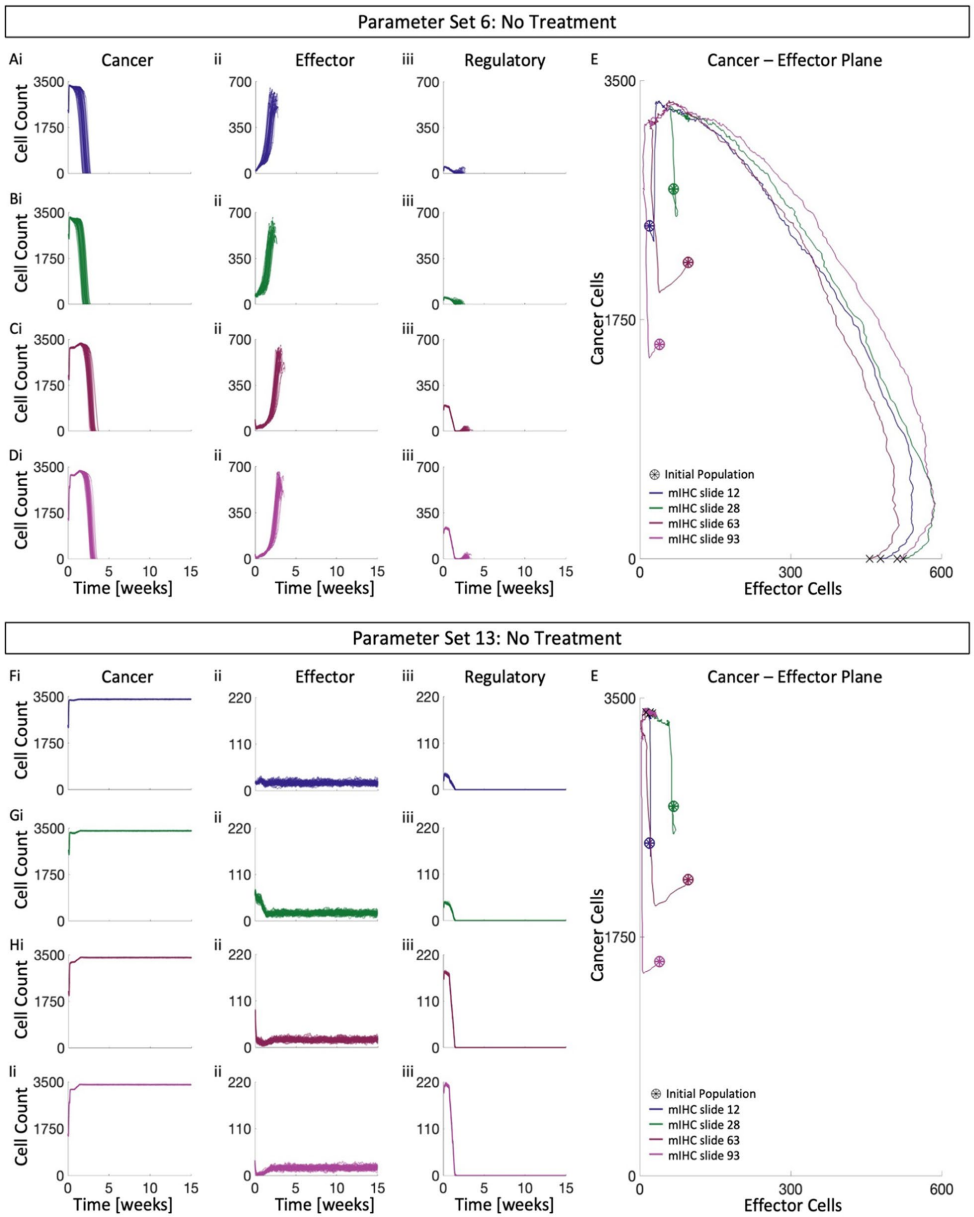
ABM recapitulates distinct tumor outcomes without treatment. Temporal dynamics of i. cancer population, ii. effector population, and iii. regulatory population, for mIHC slides **A** 12, **B** 28, **C** 63, and **D** 93, using parameter set 6. **E** Representative trajectories for A-D on the cancer-effector plane. Temporal dynamics of i. cancer population, ii. effector population, and iii. regulatory population, for mIHC slides **F** 12, **G** 28, **H** 63 and **I** 93, using parameter set 13. **J.** Representative trajectories for F-I on the cancer-effector plane. See Fig. S1 for details of mIHC slides

We generated 15 parameter sets using Latin Hypercube Sampling, each consisting of a rate of (i) recruitment of effector-immune cells due to cancer cell apoptosis (${\zeta }_{apoptosis}$), (ii) recruitment of effector-immune cells due to effector-cell induced cancer cell death (${\zeta }_{effector}$), (iii) the probability of placing an effector cell (recruited in (ii)) near the location of cancer cell death $(\mu )$, and (iv) recruitment of regulatory cells due to cancer cell proliferation $({\zeta }_{regulatory})$ (see table S4). To select plausible parameter sets, simulations without treatment were performed using each digitized mIHC slide as initial condition for the ABM (Fig. S1). Two distinct outcomes were observed: tumor elimination (e.g. parameter set 6, Fig. 3A–E) and immune escape (e.g. parameter set 13, Fig. 3F–J). These dynamics can also be visualized by plotting the cancer and effector populations against each other, highlighting the two observed behaviors (Fig. 3E, J). Despite initial tumor growth, in the case of tumor elimination the cancer-effector trajectories shift ever rightwards, indicating increases in the effector populations leading to tumor eradication (Fig. 3E). However, the same phenomenon is not observed in the case of tumor escape, suggesting that the observed tumor clearance is immune mediated. Thus, the ABM simulated immune-mediated tumor elimination and immune escape as described in literature [65]. These results suggest that there may be two distinct basins of attraction in the tumor immune ecosystem, which consist of all TIME compositions that lead to the described outcome—either immune-mediated tumor eradication (IMTE) or tumor escape. Furthermore, the existence, shape and size of these regions depend on the immune recruitment parameters [66]. Two of the 15 generated parameter sets result in moderate to complete clearance of replicates for each mIHC slide (Fig S3). Cursory analysis of the parameter space reveals no clear separation for the behavior observed (Fig. S4). In line with literature [67], we assume clinically detectable tumors have evaded the immune system, and so we discard parameter sets that lead to tumor elimination without therapy. For the rest of the manuscript, we investigate how we might use WTRT or SFRT-GRID to shift tumor trajectories from the region of immune escape to that of IMTE and elucidate the mechanisms of success for each therapy.

### WTRT does not lead to clearance with solely radiation-induced cytotoxicity

**Fig. 4:**
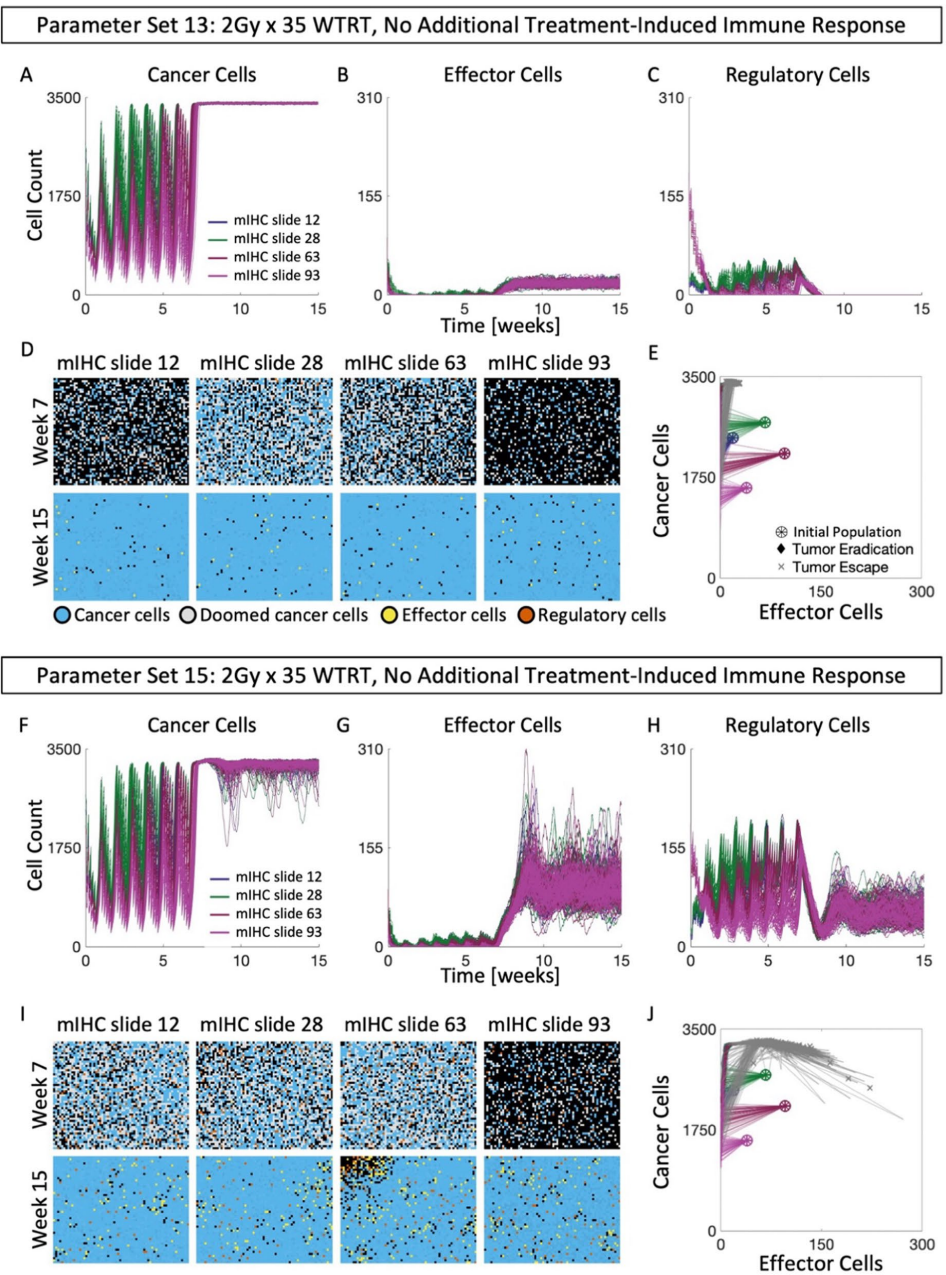
WTRT does not lead to clearance with solely radiation-induced cytotoxicity. For parameter set 13: **A–C** Temporal dynamics of each cell population of mIHC slides 12, 28, 63 and 93, when treated with 2 Gy × 35. **D** Snapshots of representative simulations of all mIHC slides at the end of weeks 7 and 15. **E** Cancer-effector cell plane of all mIHC slides treated with 2 Gy × 35 WTRT. For parameter set 15: **F–H** Temporal dynamics of each cell population of mIHC slides 12, 28, 63 and 93, when treated with 2 Gy × 35. **I** Snapshots of representative simulations of all mIHC slides at the end of weeks 7 and 15. **J.** Cancer-effector cell plane of all mIHC slides treated with 2 Gy × 35 WTRT using parameter set 15. See Fig. S1 for details of mIHC slides

We simulated radiation treatment on the digitized mIHC slides with $2\,\text{Gy} \times 35$ whole tumor radiotherapy (WTRT) using two of the 15 previously generated parameter sets (sets 13 and 15). Here, the effect of WTRT is solely lethal DNA damage. Despite early reductions in tumor burden, no tumor eradication was observed (Fig. 4A). During treatment, effector immune populations remain at or below baseline levels, and do not recover even after treatment has ended (Fig. 4B). Snapshots of a representative replicate of each tissue are shown at the end of treatment (week 7), and the end of the simulation (week 15) (Fig. 4D). It is clear that for parameter set 13, treatment with $2\,\text{Gy} \times 35$ WTRT fails to shift either of the four tissues into the region of attraction for IMTE (Fig. 4E). The immune parameter rates of set 15 lead to more intra-replicate heterogeneity of each mIHC slide, as well as higher immune populations post-treatment (Fig. 4F–I). However, despite higher effector populations, treatment again fails to shift trajectories into the region of IMTE (Fig. 4J). Thus, WTRT with solely radiation-induced cytotoxicity does not lead to clearance. However, head and neck cancers report five-year local response rates of 70–100% [68], which motivates the analysis of additional biological consequences, such as immune activation, after radiation.

### WTRT needs interaction with immune system for success

**Fig. 5:**
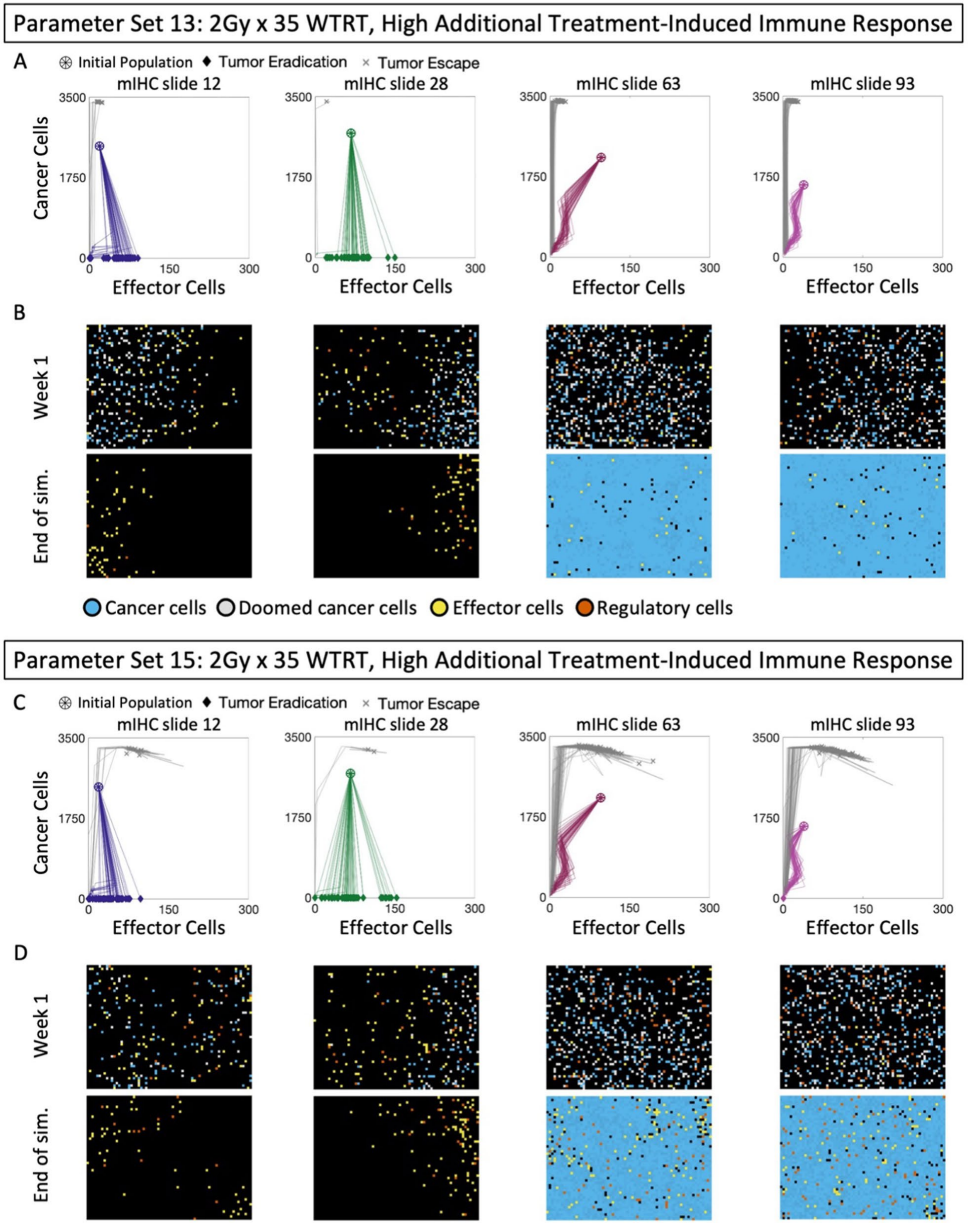
WTRT leads to clearance when Tx is sufficiently immunogenic. Parameter set 13: **A** Cancer-effector plane dynamics of mIHC slides 12, 28, 63, 93 treated with 2 Gy × 35 WTRT. **B** Snapshots of mIHC slides 12, 28, 63, 93 at the end of the first week of treatment (top row) and at the end of simulation (bottom row). Parameter set 15: **C** Cancer-effector plane dynamics of mIHC slides 12, 28, 63, 93 treated with 2 Gy × 35 WTRT. **D** Snapshots of mIHC slides 12, 28, 63, 93 at the end of the first week of treatment (top row) and at the end of simulation (bottom row)

We investigate whether a low- or highly immunogenic response to lethal DNA-damage is sufficient to obtain 70–100% tumor control (see Methods). Low immunogenicity of lethal DNA damage does not result in tumor eradication for either parameter set (Fig. S5). A highly immunogenic response to lethal DNA damage leads to eradication of the majority of trajectories of mIHC slides 12 and 28 (TE_12_ = 84% and TE_28_ = 98%), but not mIHC slides 63 and 93 (TE_63_ = 0% and TE_93_ = 0%) (Fig. 5A). Representative snapshots of the mIHC slides at the end of the first week of treatment (Fig. 5B *top row*) and the end of simulation **(**Fig. 5B *bottom row*) highlight the differential responses to WTRT (Fig. 5B first two columns: clearance vs Fig. 5B last two columns: recurrence). In the latter cases, WTRT shifts trajectories leftwards, indicating the induction of treatment related suppression of the effector populations. This phenomenon and its association with poor prognoses was recently reported in literature [69]. Parameter set 15 results in similar outcomes (TE_12_ = 82%, TE_28_ = 96%, TE_63_ = 0%, TE_93_ = 2%), but results in higher effector populations and increases intra-trajectory heterogeneity (Fig. 5C, D). Thus, $2\,\text{Gy} \times 35$ WTRT can shift trajectories of mIHC slides 12 and 28 into the IMTE region for parameter sets 13 and 15 when treatment-induced cell death is sufficiently immunogenic. However, it is insufficient to shift trajectories of mIHC slides 63 and 93 into the IMTE region.

### SFRT can be successful in contexts of high immunogenicity of DNA damage

**Fig. 6:**
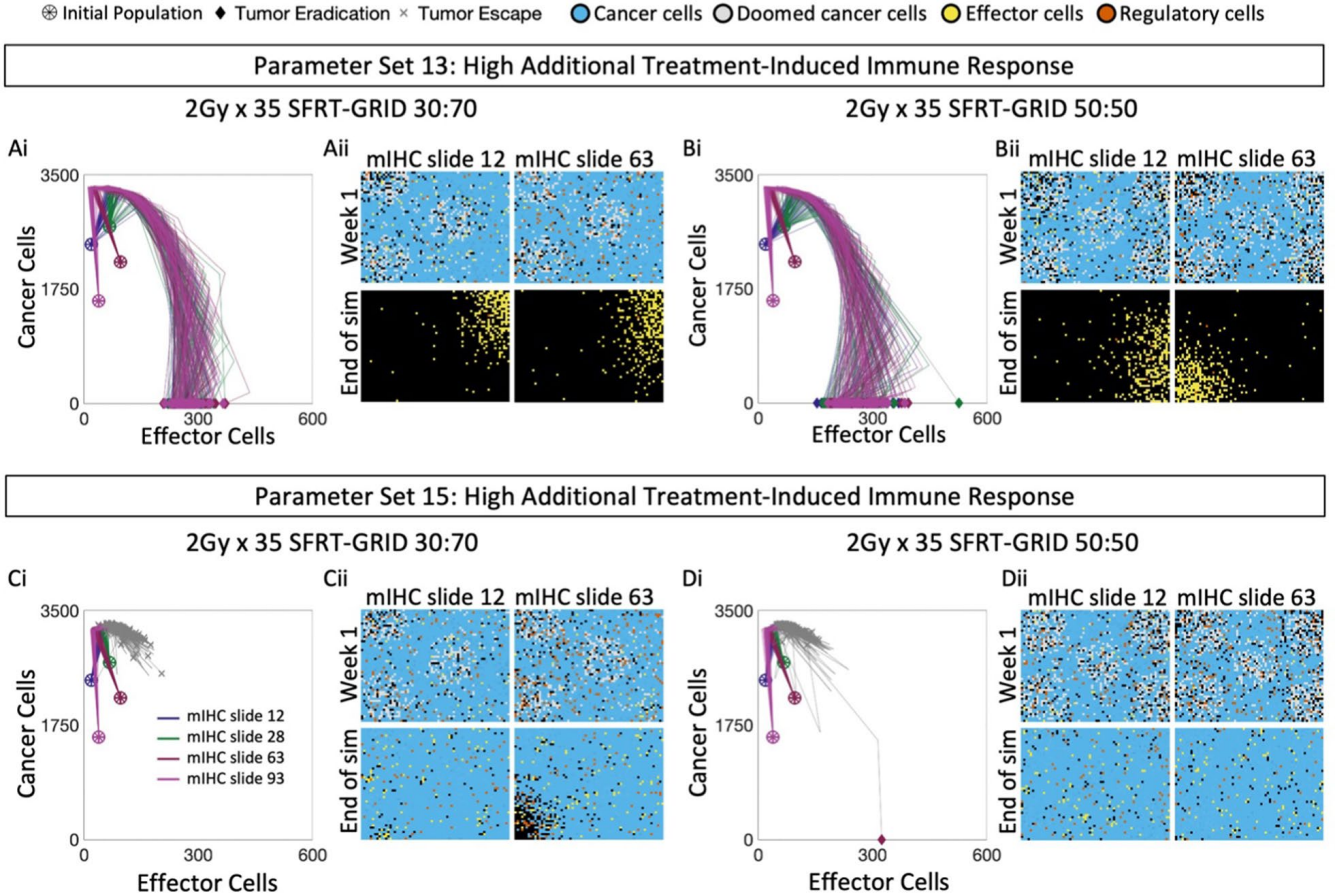
SFRT-GRID leads to clearance when Tx is sufficiently immunogenic. Parameter set 13: **A.i.** Cancer-effector plane dynamics and snapshots of mIHC slides 12 and 63 treated with 2 Gy × 35 SFRT-GRID (30:70). **A.ii.** Snapshots of representative simulations of mIHC slides 12 and 63 at the end of week 1 (top row), and tumor clearance (bottom row). **B.i.** Cancer-effector plane dynamics and snapshots of mIHC slides 12 and 63 treated with 2 Gy × 35 SFRT-GRID (50:50). **B.ii.** Snapshots of representative simulations of mIHC slides 12 and 63 at the end of week 1 (top row), and tumor clearance (bottom row). Parameter set 15: **C.i.** Cancer-effector plane dynamics and snapshots of mIHC slides 12 and 63 treated with 2 Gy × 35 SFRT-GRID (30:70). **C.ii.** Snapshots of representative simulations of mIHC slides 12 and 63 at the end of week 1 (top row), and week 15 (bottom row). **D.i.** Cancer-effector plane dynamics and snapshots of mIHC slides 12 and 63 treated with 2 Gy × 35 SFRT-GRID (50:50). **D.ii.** Snapshots of representative simulations of mIHC slides 12 and 63 at the end of week 1 (top row), and week 15 (bottom row). See Fig. S7 for snapshots of mIHC slides 28 and 93

We next asked whether SFRT could recapitulate the response rates of mIHC slide 12 and 28 above, while improving response rates of those tumors for which WTRT was unsuccessful. To this end, we administered $2\,\text{Gy} \times 35$ of either SFRT-GRID (30:70) or SFRT-GRID (50:50) to all four mIHC slides, with the assumption that Tx is immunogenic. For parameter set 13, treatment with either SFRT-GRID geometry is sufficient to shift all trajectories of mIHC slides 12, 28, 63 and 93 into the immune-mediated tumor eradication region (Fig. 6A, B, Fig. S7A, B). Interestingly we see that the first week of treatment induces an upwards and right shift for slides 12 and 28, indicating early increases in both effector and cancer populations, but slides 63 and 93 experience an initial upwards and left shift indicating an increase in cancer cells and a decrease in effector cells. Subsequent weeks of treatment generally shift the tumor trajectories downwards and to the right, indicating an increasing effector population. However, when using parameter set 15 no models exhibit notable tumor eradication. Instead, trajectories shift within the tumor escape region, remaining at high levels of cancer cells (Fig. 6C, D, Fig. S7C, D). Thus, for certain pre-treatment state and parameter set combinations SFRT-GRID successfully shifts trajectories into the tumor-eradication region, and is equally or more effective than WTRT despite delivering less total average dose ($0.81\,\text{Gy}$ and $1.15\,\text{Gy}$ average dose per fraction for SFRT-GRID(30:70) and SFRT-GRID (50:50) respectively vs $2\,\text{Gy}$ for WTRT).

### Elucidating the biological mechanism of tumor response

In the following subsections we interrogate the mechanisms of the observed outcomes following treatment with WTRT and SFRT-GRID, using representative slide-parameter set pairs for each treatment type. Specifically, for WTRT we use mIHC slide 12 and parameter set 15, while we use mIHC slide 63 and parameter set 13 for SFRT-GRID.

### Treatment-induced DNA damage is the primary mechanism of tumor response to WTRT

**Fig. 7:**
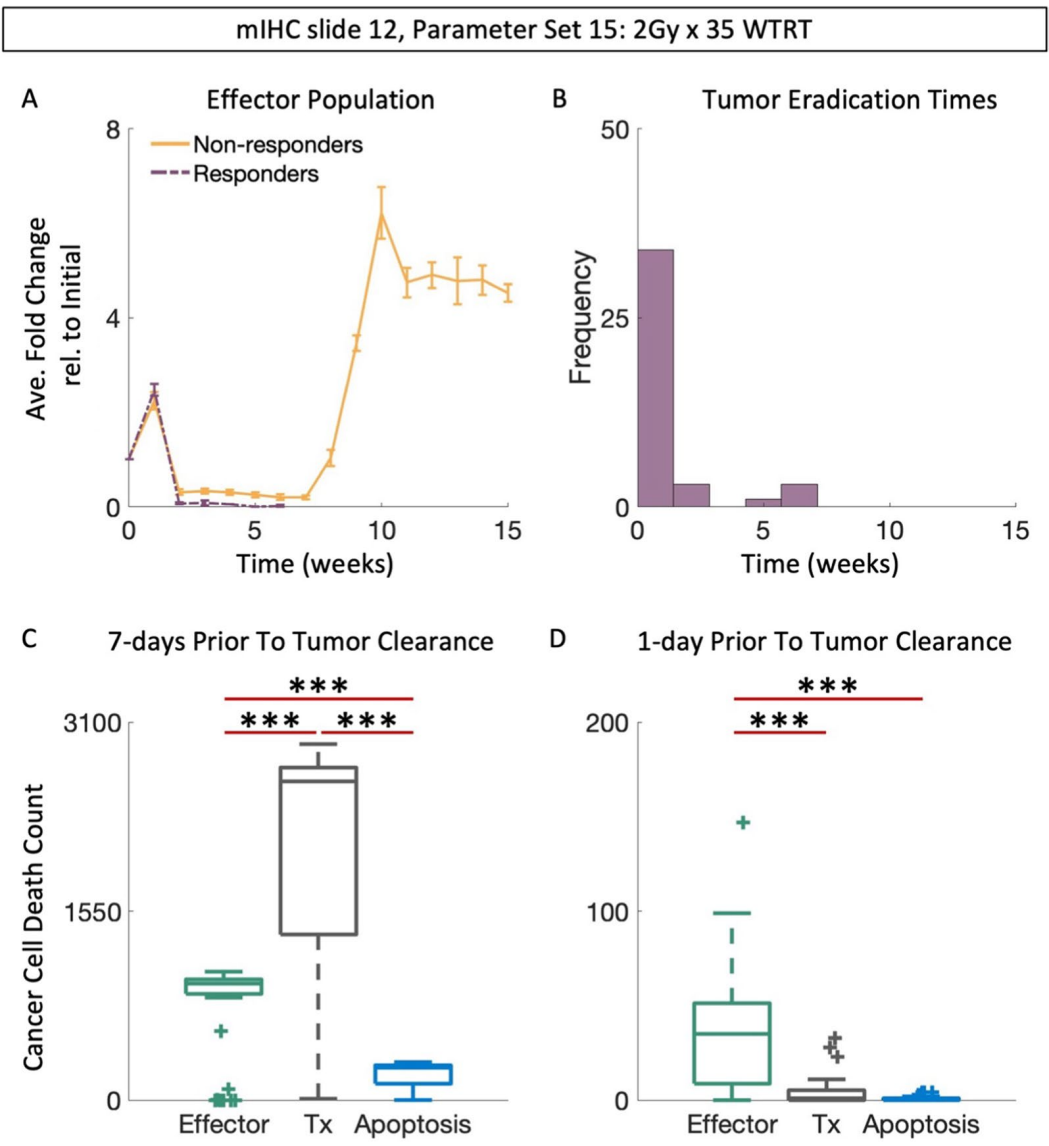
WTRT success relies on maximizing log-cell kill. **A** Average fold change of effector population in responding or non-responding mIHC slide 12 tumors treated with 2 Gy × 35 of WTRT. **B** Distribution of clearance times for responding tumors in A. ** C**, **D** Contribution of effector-mediated cancer cell death, treatment, and apoptosis in the 7 day or 1 day period leading up to and including clearance of tumors treated with WTRT respectively. Error bars are mean $\pm$ SEM. (*** *p* < 0.001, two-sided Wilcoxon rank sum test, Table S7)

When using parameter set 15, the first week of treatment with WTRT results in notable increases in the average effector population for mIHC slide 12. Subsequent applications of WTRT lead to sustained effector suppression, which only wears off weeks after treatment has ended (Fig. 7A). Interestingly, the average effector population of responding simulations are higher than non-responders following the first week of treatment, but lower than non-responders during subsequent weeks (Fig. 7A*, solid vs dashed curves*). This initial difference corresponds to the time-period in which most clearances occur (Fig. 7B), suggesting that WTRT reduces the tumor burden either completely or to such a degree that the suppressed effector population can eliminate the remaining cancer cells. The dominant mechanism of cancer cell death within the 7-day period leading up to and including tumor eradication is treatment induced DNA damage (Fig. 7C, Table S7, *p* < *0.005*), while effector-mediated cell death is the primary mechanism during the 24-h period leading up to tumor clearance (Fig. 7D, Table S7, *p* < *0.005*). Thus, DNA damage contributes more to tumor clearance during WTRT than effector mediated cancer cell death.

### SFRT-GRID promotes an accumulation of effector cells

**Fig. 8:**
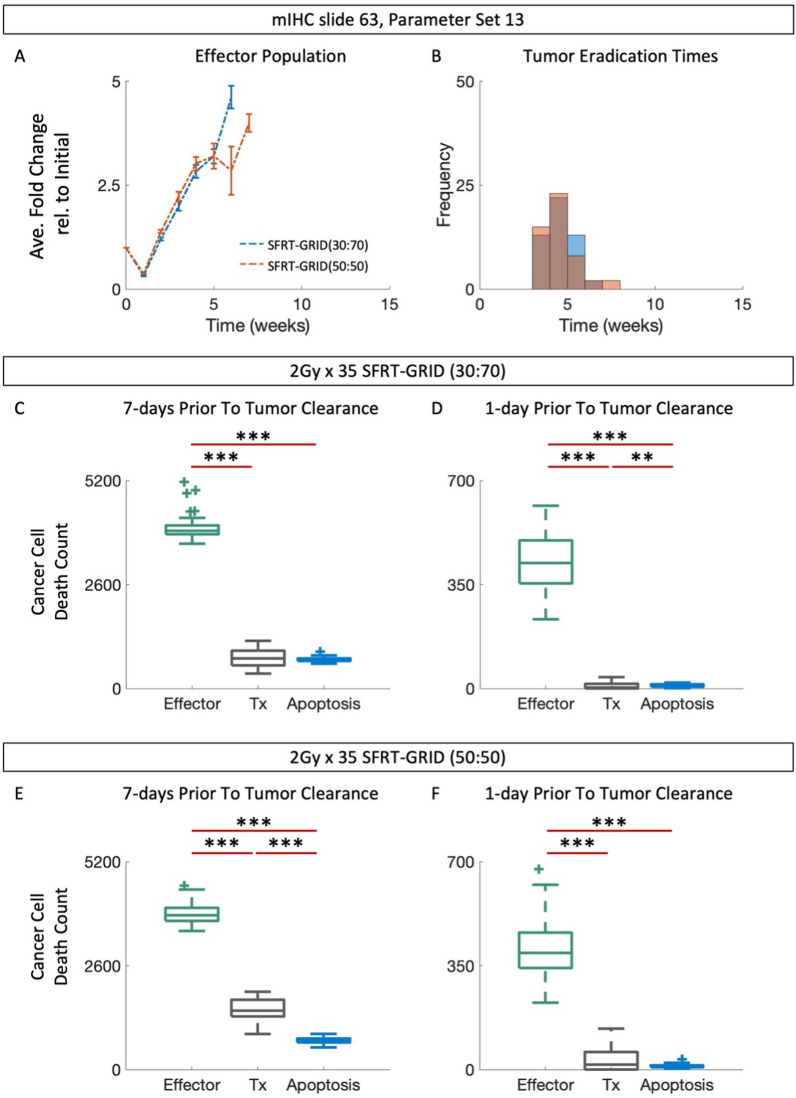
SFRT potentiates immune mediated tumor clearance.** A** Average fold change of effector population in responding or non-responding mIHC slide 63 tumors treated with 2 Gy × 35 of WTRT, SFRT-GRID(30:70) or SFRT-GRID(50:50). **B** Distribution of clearance times for responding tumors in (A). **C**,** D** Contribution of effector-mediated cancer cell death, treatment, and apoptosis in the 7 day or 1 day leading up to and including clearance of tumors treated with SFRT-GRID(30:70). **E**, **F** Contribution of effector-mediated cancer cell death, treatment, and apoptosis in the 7 day or 1 day leading up to and including clearance of tumors treated with SFRT-GRID(50:50). Error bars are mean $\pm$ SEM. (**p < 0.01, ***p < 0.001, two-sided Wilcoxon rank sum test, Table S8–S9)

Different effector cell dynamics are observed when we use parameter set 13. During the first week of treatment with either SFRT-GRID geometry we see a large decline in the average fold change of the effector population of mIHC slide 63. However, subsequent weeks of treatment result in a cumulative increase in the effector population (Fig. 8A, *dashed curves*).. The distribution of clearance times of tumors treated with SFRT-GRID(30:70) or SFRT-GRID (50:50) suggest that the observed accumulation of effector cells may be responsible for tumor eradication (Fig. 8B).

### Cumulative immunogenic cell death during SFRT-GRID drives cancer cell clearance

To confirm this, we determined the dominant mechanism of cancer cell death (effector-cell mediated, treatment-induced, or apoptosis) within the 7 day and 24-h periods leading up to and including the timestep in which tumor clearance occurred for mIHC slide 63. For tumors treated with either of the SFRT-GRID geometries, effector cell mediated cancer cell death is the primary mechanism (Fig. 8C-F, Table S8, S9, *p* < *0.005*). Taken together, these data suggest that cumulative immunogenic cell death during SFRT-GRID drives cancer cell clearance, and thus SFRT-GRID, but not WTRT, may potentiate immune-mediated tumor control (Fig. 8).

## Discussion

We developed an ABM to study the biological and immunological consequences of radiation therapy as a function of pre-irradiation TIME composition using immunohistochemistry slides, spatial radiation distribution, and model parameters. The goal of this study was to explore the immunological consequences of whole tumor and spatially fractionated radiotherapy. In this hypothesis-generating approach, we explored the parameter space to arrive at parameter combinations that yield biologically and clinically feasible population-level dynamics. While we focus on HNC, the model is not calibrated nor validated for a specific cancer type.

Unrepairable, lethal DNA damage was long thought to be the only driving factor of the tumoricidal effect of radiotherapy. In the described model, all simulations of whole tumor radiation with generic radiosensitivity parameters did not lead to tumor extinction despite the very low number of cancer cells at simulation initialization compared to clinical tumors (we only consider the cells counted in the biopsy tissue, not the entire clinical target volume). While there is a significant loss of cancer cells early during radiation, cancer cell repopulation appears to outcompete radiation-induced cell death in later stages of therapy. This is a visualization of accelerated repopulation during radiation therapy, and one motivation for hyperfractionated radiation accelerated treatment, which is most helpful later in treatment [70].

Of importance here, whole tumor radiation also wipes out nearly all immune cells. The addition of immunologic cell death and radiation-induced recruitment of additional immune cells was able to simulate tumor control comparable to clinical observations. This adds to the growing body of literature that stresses the immune-related effect of radiation and warrants further exploration into the biological and especially immunological consequences of radiation, and how to best tailor radiation towards immune activation compared to historical log-cell kill maximization. In the setting of strong radiation-induced anti-tumor immunity, we see that spatially fractionated radiation synergizes with the immune system, and may create areas of low dose radiation where immune cells are sheltered and can amount a robust attack on cancer cells outside of the radiation fields. While these results confirm our tested hypothesis, other herein untested parameter combinations and biological and radiobiological mechanisms may also be at play in providing whole tumor radiation control, such as re-oxygenation and re-sensitization of cancer cells to subsequent radiation fractions. The plethora of nonlinear radiation response mechanisms motivates a rigorous analysis of this biological complexity to guide future pre-clinical and clinical experimentation to fully decipher the importance of radiation-induced antitumor immunity.

In our study, we set out to test the immunological consequences of spatially fractionated radiation compared whole tumor radiation over a seven-weeks course of radiotherapy. In the clinic, SFRT-GRID is currently applied as an upfront ablative fraction of $15\,\text{Gy} \times 1$ followed by whole tumor radiation with $2\,\text{Gy} \times 25$ [19, 20]. Comparison of the treatment efficacy for each mIHC slide when parameter set 13 is used shows that the clinical ablative SFRT-GRID schedule is *less* effective for mIHC slides 63 and 93 than the fractionated SFRT-GRID schedules. However, for parameter set 15, we observe that the ablative schedules are more effective for certain mIHC slides (Table S10). Analysis of the underlying mechanism highlights the differences in underlying mechanisms of action: fractionated SFRT-GRID synergizes with the immune effector population, while ablative SFRT-GRID debulks the tumor population, and suppresses the immune effector population (Fig. S10, Table S11).

These results offer a novel approach of SFRT as a sole treatment plan without subsequent whole tumor radiation as currently done in clinical practice. Of note, however, the success of SFRT is crucially dependent on pre-treatment TIME composition and model parameters. It is therefore crucial to take the findings herein and rigorously calibrate and validate the ABM for specific cancer types and clinical immune infiltration scenarios before clinical translation.

Central to developing in silico models is the need for simplifying assumptions. Through these we condense current biological knowledge, with the aim of retaining the most critical components while minimizing the complexity and inherent variability of in vitro or in vivo experiments. Here, we have assumed that the SFRT-GRID block is always positioned such that the placement of the openings aligns perfectly throughout the course of treatment, thereby conserving the initial heterogeneous dose distribution. In reality, tumor motion due to respiratory or other physiological movements, may cause intra-fraction dose smearing. In addition, slight differences in patient alignment or tumor size and shape may result in inter-fraction changes of the dose distribution. This blurring could conceivably lead to higher cumulative valley doses, and less contrast between peaks and valleys. The immune activation / enhancement seen during SFRT may consequently be curbed. While accurate tumor tracking and patient alignment are essential to maintain the integrity of the peak and valley dose distributions, research suggests that dose smearing may be minimized by selecting appropriate SFRT-GRID block designs and orienting the blocks according to the path of tumor motion [71].

Prior to experimental validation of the hypotheses presented here, murine scale SFRT-GRID must be manufactured. While replicating the SFRT-GRID geometries used in this study may not be feasible, replicating the open-to-shielded ratios is easily feasible. Of note, the focus of this work is not to design SFRT-GRID blocks for translation into preclinical experimentation; rather, we aimed to investigate if such experimentation is theoretically warranted.

We used multiplex immunohistochemistry (mIHC) stained tissue samples as initial conditions for the ABM to simulate radiation on realistic TIME states. While pre-treatment mIHC tissues allow us to visualize the cellular TIME makeup, tissues are unavailable in a longitudinal manner, and little to no information is available regarding the dynamics that give rise to the TIME state at that point in time. In the future, novel machine learning approaches may be able to help decipher ABM parameters that lead to specific TIME compositions as seen in patient biopsies [72].

## Conclusion

Radiation-induced anti-tumor immunity seems pivotal in eradicating tumors. Thus, radiation should be tailored to each patient’s tumor immune ecosystem to eradicate cancer cells, protect immune cells, and harness the synergy with the immune system. For some patients, spatially fractionated radiation rather than whole tumor radiation may be a promising approach. To prospectively identify who would benefit from SFRT, further research must be dedicated to rigorous calibration and validation of the presented modeling approach.

## Supplementary Information

Supplementary Material 1

## Data Availability

All de-identified post-processed clinical data, as well as simulation code (Java in HAL environment: link https://halloworld.org/) and statistical analysis code (MATLAB) will be deposited in a github repository.

## Abbreviations

ABM: Agent-based model
DAMPS: Damage associated molecular patterns
HMGB1: HIGH mobility group box 1
HNC: Head and neck cancer
HNSCC: Head and neck squamous cell carcinoma
IL-6: Interleukin 6
IMTE: Immune-mediated tumor elimination
mIHC: Multiple immunohistochemistry
NF-κB: Nuclear factor kappa-light-chain-enhancer of activated B cells
PD-1: Programmed cell death protein 1
PDL1: Programmed death ligand 1
PVDR: Peak-valley dose ratio
RT: Radiotherapy/radiation
SF: Survival fraction
SFRT: Spatially fractionated radiotherapy
SFRT-GRID: Spatially fractionated radiotherapy administered using GRID blocks
TIME: Tumor immune microenvironment
TLR2: Toll-like receptor 2
TLR3: Toll-like receptor 3
TLR4: Toll-like receptor 4
TLR9: Toll-like receptor 9
TNF-α: Tumor-necrosis factor-alpha
Tx: Treatment
WTRT: Whole tumor radiotherapy
