# Supplementary material for: Spatially fractionated GRID radiation potentiates immune-mediated tumor control
Source: Radiat Oncol. 2024 Sep 13;19:121. doi: 10.1186/s13014-024-02514-6 (PMC11401399; doi:10.1186/s13014-024-02514-6)
Supplement: Supplementary file 1 — Supplementary Material 1 [file 13014_2024_2514_MOESM1_ESM.docx]

**Spatially Fractionated GRID radiation potentiates immune-mediated tumor control.**

Rebecca A. Bekker^1,4^, Nina Obertopp^2,4^, Gage Redler^3^, José Penagaricano^3^, Jimmy J. Caudell^3^, Kosj Yamoah^3^, Shari Pilon-Thomas^2^, Eduardo G. Moros^3^, Heiko Enderling^5,6,*^

^1^Department of Integrated Mathematical Oncology, H. Lee Moffitt Cancer Center & Research Institute, Tampa, FL, 33612, USA

^2^Department of Immunology, H. Lee Moffitt Cancer Center & Research Institute, Tampa, FL, 33612, USA

^3^Department of Radiation Oncology, H. Lee Moffitt Cancer Center & Research Institute, Tampa, FL, 33612, USA

^4^Cancer Biology Ph.D. Program, University of South Florida, Tampa, FL, 33612, USA

^5^ Department of Radiation Oncology, The University of Texas MD Anderson Cancer Center, Houston, TX, 77030, USA

^6^Institute for Data Science In Oncology, The University of Texas MD Anderson Cancer Center, Houston, Texas, 77030, USA

^*^ Correspondence: [henderling@mdanderson.org](mailto:henderling@mdanderson.org)

**SUPPLEMENTARY FIGURES AND TABLES**

| Antibody | Target | Description |
| --- | --- | --- |
| CD3 | Cluster of Differentiation 3 | T lymphocytes |
| CD4 | Cluster of Differentiation 4 | “Helper” T cells, a subset of T lymphocytes |
| CD8 | Cluster of Differentiation 8 | Cytotoxic T cells, a subset of T lymphocytes (Effector cells) |
| FOXP3 | Forkhead Box P3 | Treg cells, a subset of T lymphocytes (Regulatory cells) |
| PCK | Pan-Cytokeratin | Stains for cytokeratins, a structural protein in epithelial cells, used to identify cancer cells of epithelial origin |
| DAPI | 4',6-diamidino-2-phenylindole | DNA / cell nuclei |

**Table S1:** Overview of stains used in multiplex immunohistochemistry


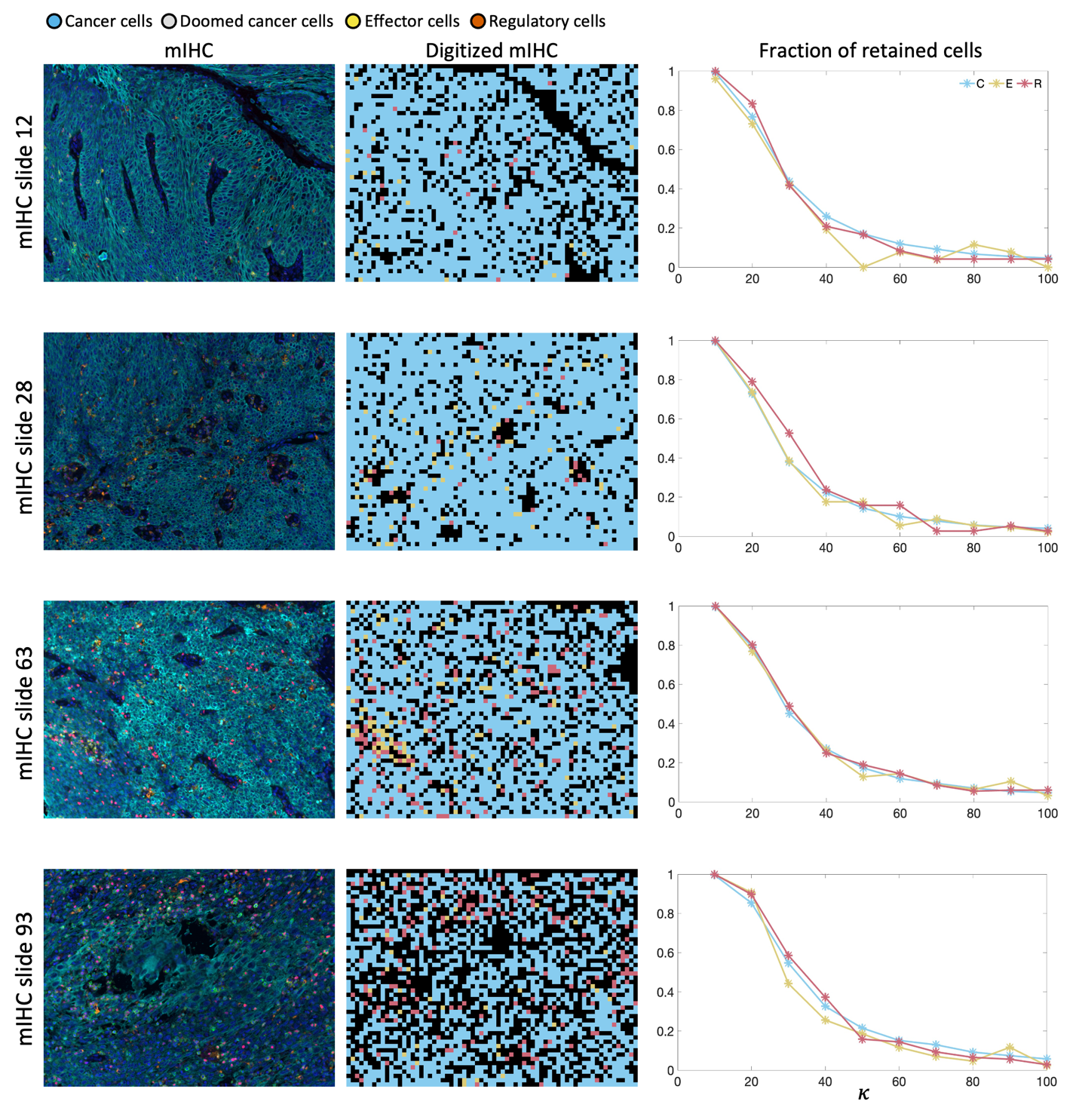


**Fig. S1** Multiplex immunohistochemistry slides (left), associated digitized mIHC /*in silico* tumors (center), and the fraction of retained cells of each subpopulation of each *in silico* tumor, for conversion factors $\kappa\in\left[ 10,100 \right]$.


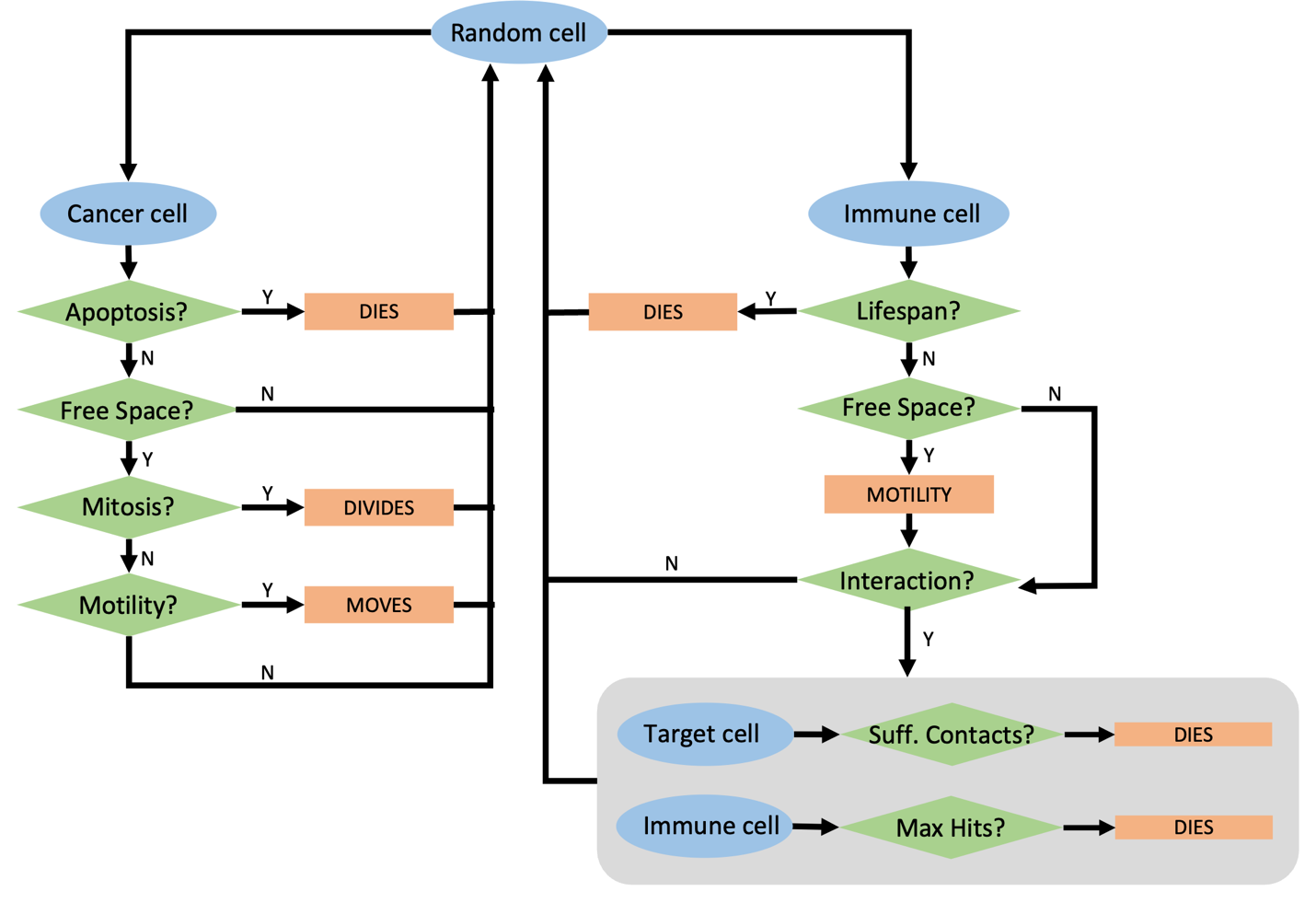


**Fig. S2** *In silico* rules governing cancer cell and immune cell behavior.

| **Name** | **Value** | **Units** | **References** |
| --- | --- | --- | --- |
| Cell cycle duration | $x\sim N\left( 24,2 \right),$  $x \epsilon[18,30]$ | $hr$ |  |
| Random death probability | 0.003 | $hr^{-1}$ | (1) |
| Migration speed | 6 | $bodylengths /hr$ | (2, 3) |
| Effector cell contacts needed for death | 3 |  | (4) |
| Repair time of contact from effector cell | 1 | $hr$ | (5) |
| $SF_{C}(2Gy)$ | 0.48 |  |  |

**Table S2.** Cancer cell parameters.

| **Name** | **Value** | **Units** | **Reference** |
| --- | --- | --- | --- |
| Lifespan | 10 | $days$ | (6) |
| Maximum number of cytotoxic contacts with cancer cells | 10 |  |  |
| Regulatory cell contacts needed for death | 3 |  |  |
| Migration speed | $15$ | $bodylengths /hr$ | (7-9) |
| Recruitment due to cancer cell random death | $\zeta_{Apoptosis}$ | $cells / event$ | **LHS** |
| Recruitment due to effector induced cancer cell death | $\zeta_{effector}$ | $cells / event$ | **LHS** |
| Recruitment due to radiation induced cancer cell death | $\zeta_{Tx}$ | $cells / event$ | **LHS** |
| Placement of immune cells | $\mu$ |  | **LHS** |
| $SF_{E}(2Gy)$ | $\approx0.60$ |  | (10) |

**Table S3.** Effector cell parameters. LHS: Latin hypercube sampling. Parameters with LHS denoted as a reference were generated using LHS as outlined in the methods.



| **Name** | **Value** | **Unit** | **Reference** |
| --- | --- | --- | --- |
| Life span | 10 | $days$ |  |
| Max contacts with prey | 10 |  |  |
| Migration speed | 15 | $\frac{bodylengths}{hr}$ |  |
| Recruitment due to cancer cell birth | $\zeta_{regulatory}$ | $\frac{cells}{event}$ | **LHS** |
| $SF_{E}(2Gy)$ | $\approx0.77$ |  | (10) |

**Table S4.** Regulatory cell parameters. LHS: Latin hypercube sampling. Parameters with LHS denoted as a reference were generated using LHS as outlined in the methods.

| **Parameter** | **Meaning** | **Range** | **Units** |
| --- | --- | --- | --- |
| $\zeta_{apoptosis}$ | Recruitment rate of effector cells due to apoptosis of cancer cells | [0.05, 0.2] | cells per cancer cell death |
| $\zeta_{immune}$ | Recruitment rate of effector cells due to effector cell-induced death of cancer cells | [0.05, 0.2] | cells per cancer cell death |
| $\mu$ | Probability of placing effector cells that are recruited due to effector cell induced cancer cell death near those locations | [0.2, 0.6] | - |
| $\zeta_{regulatory}$ | Recruitment rate of regulatory cells due to proliferation of cancer cells | [0.001, 0.2] | cells per cancer cell birth |

**Table S5.** Parameter ranges used in the Latin Hypercube Sampling to generate parameter sets 1-15.

| Parameter set | $\zeta_{apoptosis}$ | $\zeta_{effector}$ | $\zeta_{regulatory}$ | $\mu$ |
| --- | --- | --- | --- | --- |
| 1 | 0.145 | 0.065 | 0.15357 | 0.24 |
| 2 | 0.085 | 0.085 | 0.11377 | 0.45333 |
| 3 | 0.175 | 0.185 | 0.19337 | 0.34667 |
| 4 | 0.115 | 0.095 | 0.1005 | 0.26667 |
| 5 | 0.095 | 0.165 | 0.0076333 | 0.37333 |
| 6 | 0.135 | 0.155 | 0.034167 | 0.29333 |
| 7 | 0.165 | 0.105 | 0.1801 | 0.32 |
| 8 | 0.185 | 0.075 | 0.073967 | 0.21333 |
| 9 | 0.055 | 0.135 | 0.16683 | 0.56 |
| 10 | 0.155 | 0.055 | 0.047433 | 0.4 |
| 11 | 0.105 | 0.115 | 0.087233 | 0.53333 |
| 12 | 0.195 | 0.125 | 0.1403 | 0.58667 |
| 13 | 0.075 | 0.145 | 0.0209 | 0.48 |
| 14 | 0.065 | 0.175 | 0.12703 | 0.50667 |
| 15 | 0.125 | 0.195 | 0.0607 | 0.42667 |

**Table S6.** Parameter values of parameter sets 1-15. Parameter sets highlighted in green lead to tumor clearance without treatment, and parameter sets highlighted in yellow are the ones used within the manuscript.

**Fig. S3.** Simulation trajectories of mIHC slides 12, 28, 63 and 93 using parameter sets 1-15, without treatment. Parameter sets 5 and 6 consistently lead to tumor eradication. (Blue – cancer cells, yellow – effector cells, red – regulatory cells).
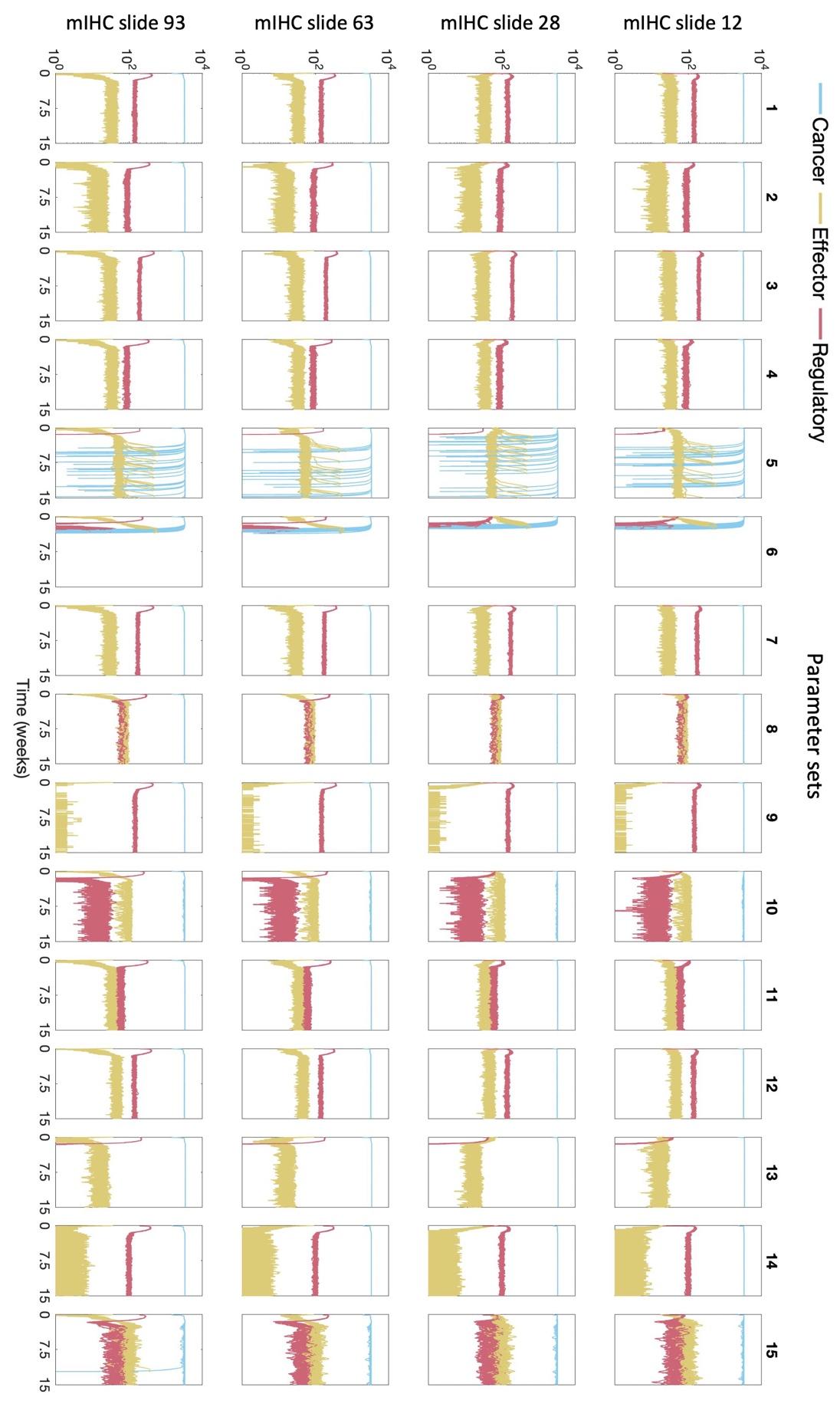


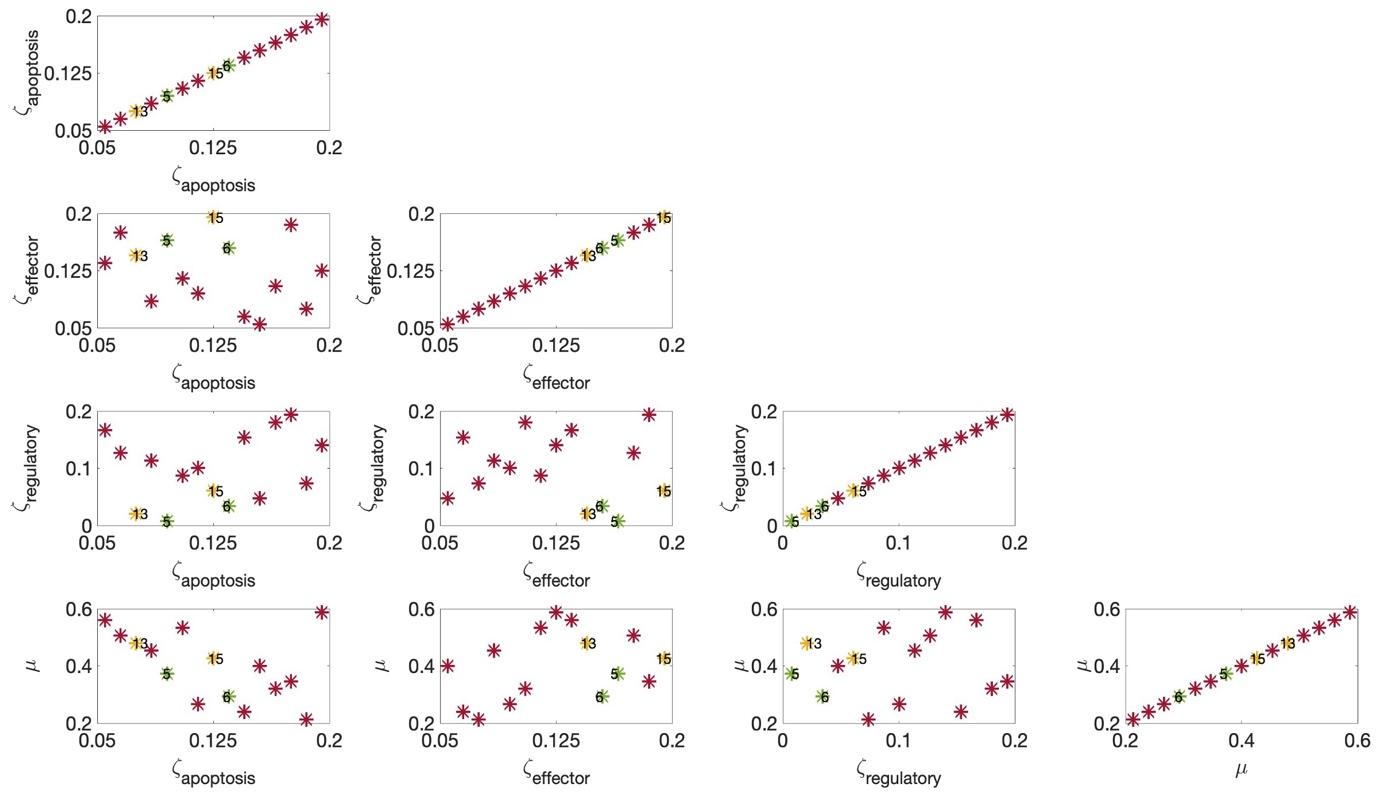


**Fig. S4** Distribution of the generated parameter sets in the explored parameter spaces: (i) $\zeta_{apoptosis}$ recruitment of effector-immune cells due to cancer cell apoptosis,

(ii) $\zeta_{effector}$ recruitment of effector-immune cells due to effector-cell induced cancer cell death, (iii) $\zeta_{regulatory}$ recruitment of regulatory cells due to the increasing cancer cell population, (iv) 𝜇 the probability of placing an effector cell (recruited in (ii)) near the location of cancer cell death, (Green stars indicating tumor eradication without treatment: parameter sets 5 and 6, yellow stars indicating the parameter sets used in this study: parameter sets 13 and 15, red stars: other parameter sets).


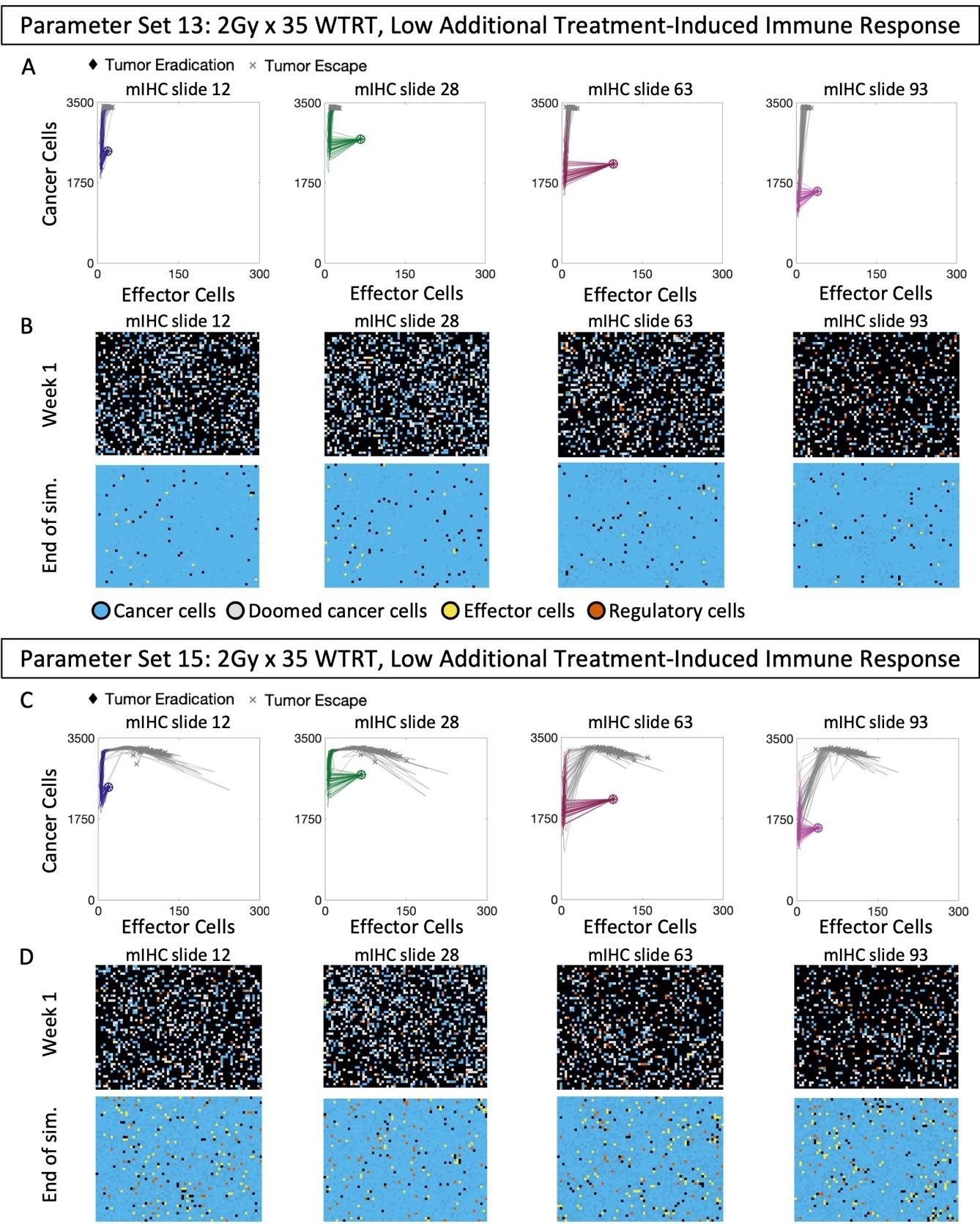


**Fig. S5 WTRT does not leads to clearance when immunogenicity of Tx is low.** Parameter set 13: **A.** Cancer-effector plane dynamics of mIHC slides 12, 28, 63, 93 treated with 2Gy x 35 WTRT, using parameter set 13. **B.** Snapshots of mIHC slides 12, 28, 63, 93 at the end of the first week of treatment *(top row)* and at the end of simulation.

Parameter set 15: **C.** Cancer-effector plane dynamics of mIHC slides 12, 28, 63, 93 treated with 2Gy x 35 WTRT, using parameter set 15. **D.** Snapshots of mIHC slides 12, 28, 63, 93 at the end of the first week of treatment *(top row)* and at the end of simulation *(bottom row).* (Legend: A,C: non-grey line segments – on treatment, grey line segments – post-treatment, diamonds – tumor eradication, crosses – tumor escape. B,D: cyan - cancer cells, grey – doomed cancer cells, with irreparable RT-induced damage, yellow - effector immune cells, red - regulatory immune cells).

On next page:

**Fig. S6 SFRT-GRID does not lead to clearance when treatment isn’t sufficiently immunogenic.** Phase plane dynamics of cancer cells and effector cells, and ABM snapshots at the end of weeks 1 and 15 of the simulation. **A.** Parameter set 13, treatment is not immunogenic, i. SFRT-GRID (30:70); ii. SFRT-GRID (50:50). **B.** Parameter set 13, treatment has low immunogenicity, i. SFRT-GRID (30:70); ii. SFRT-GRID (50:50). **C.** Parameter set 15, treatment is not immunogenic, i. SFRT-GRID (30:70); ii. SFRT-GRID (50:50). **D.** Parameter set 15, treatment has low immunogenicity, i. SFRT-GRID (30:70); ii. SFRT-GRID (50:50)


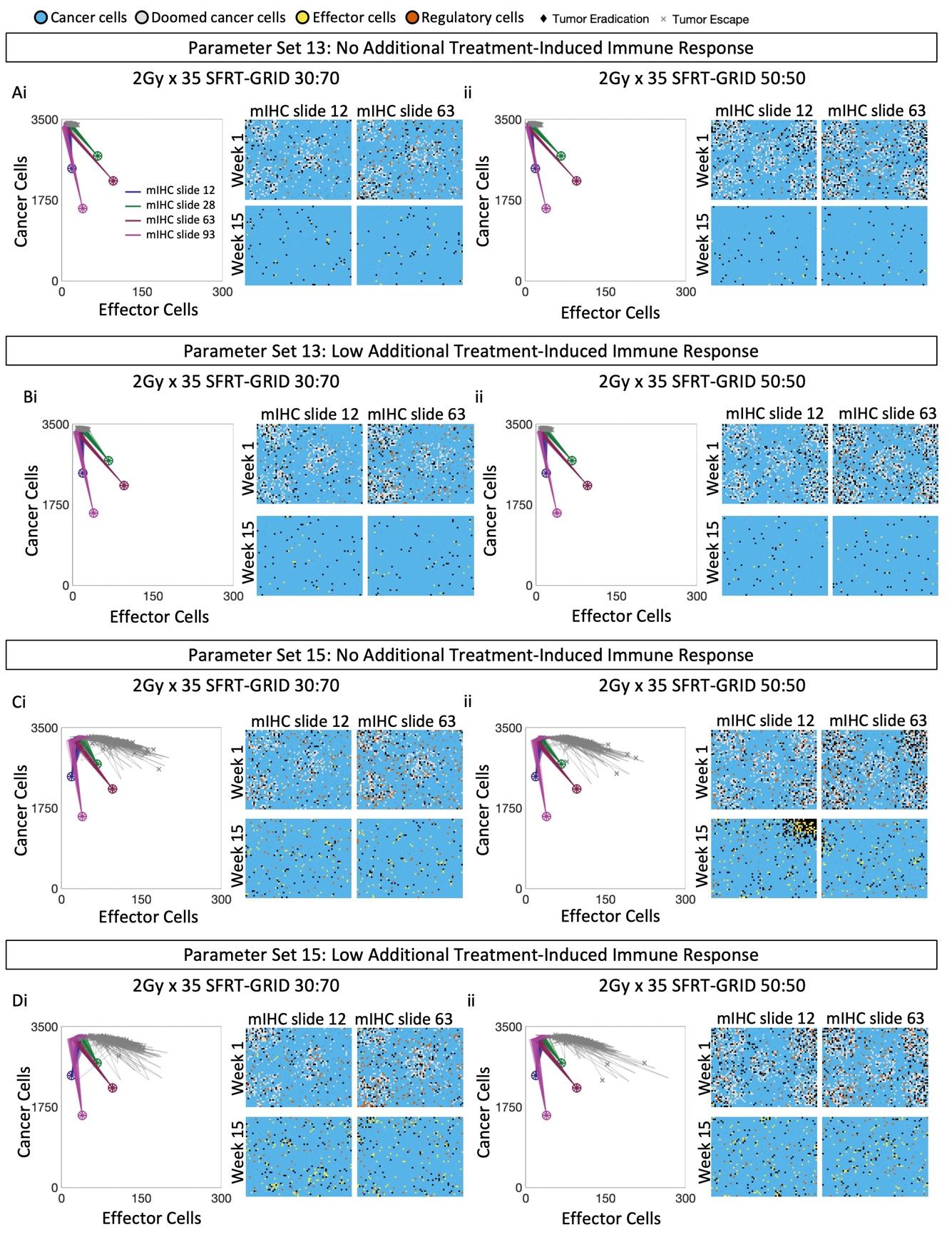


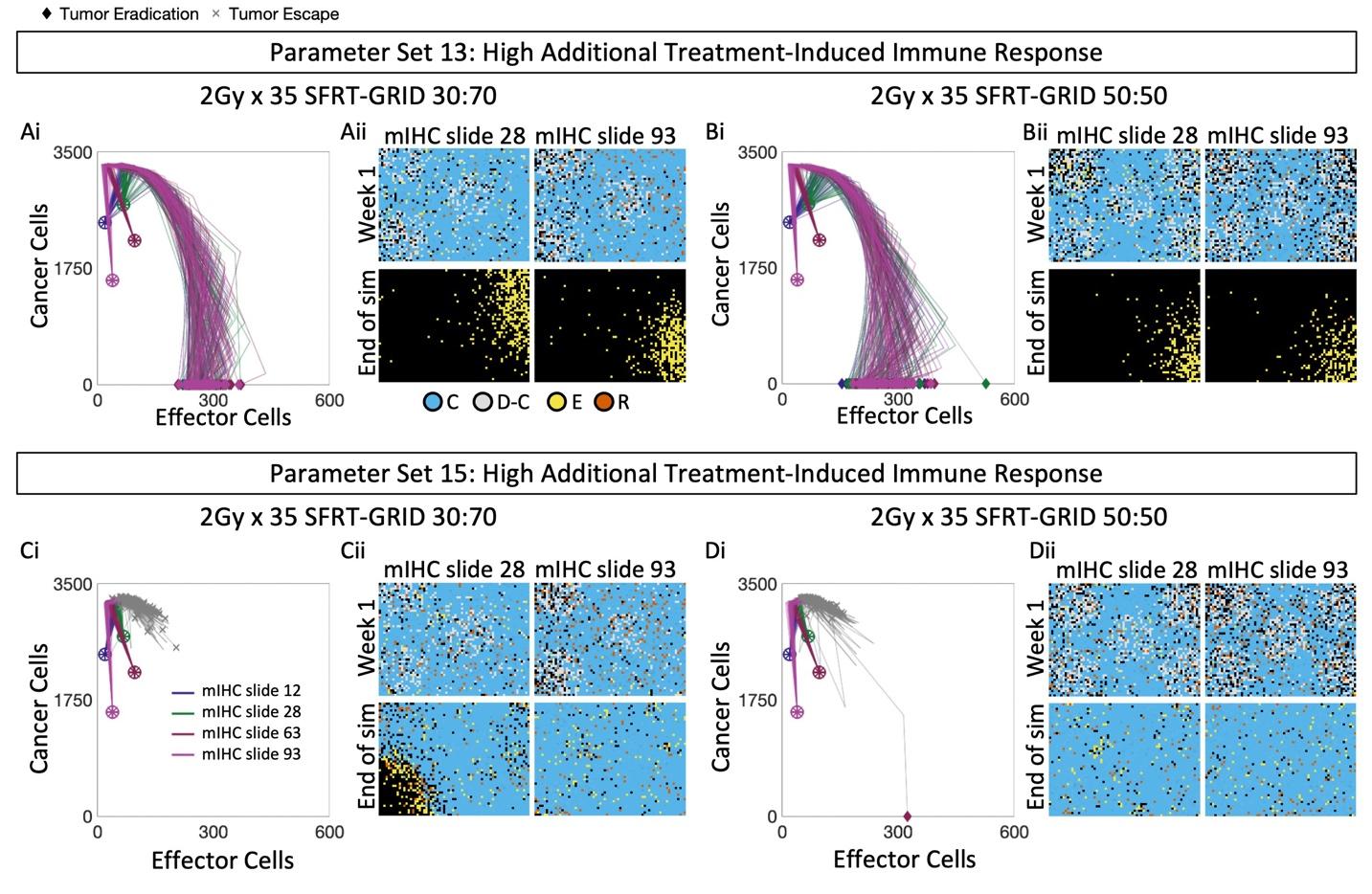


**Fig. S7 SFRT-GRID leads to clearance when Tx is sufficiently immunogenic.**

Parameter set 13: **A.i.** Cancer-effector plane dynamics and snapshots of mIHC slide 28 and 93 treated with 2Gy x 35 SFRT-GRID (30:70). **A.ii.** Snapshots of representative simulations of mIHC slides 28 and 93 at the end of week 1 *(top row)*, and tumor clearance *(bottom row).*  **B.i.** Cancer-effector plane dynamics and snapshots of mIHC slide 28 and 93 treated with 2Gy x 35 SFRT-GRID (50:50). **B.ii.** Snapshots of representative simulations of mIHC slides 28 and 93 at the end of week 1 *(top row)*, and tumor clearance *(bottom row).*

Parameter set 15: **C.i.** Cancer-effector plane dynamics and snapshots of mIHC slide 28 and 93 treated with 2Gy x 35 SFRT-GRID (30:70). **C.ii.** Snapshots of representative simulations of mIHC slides 28 and 93 at the end of week 1 *(top row)*, and week 15 *(bottom row).*  **D.i.** Cancer-effector plane dynamics and snapshots of mIHC slide 28 and 93 treated with 2Gy x 35 SFRT-GRID (50:50). **D.ii.** Snapshots of representative simulations of mIHC slides 28 and 93 at the end of week 1 *(top row)*, and week 15 *(bottom row).*

See **Fig. 6** for snapshots of mIHC slides 12 and 63.

(Legend: Cancer-Effector planes: non-grey line segments – on treatment, grey line segments – post-treatment, diamonds – tumor eradication, crosses – tumor escape. ABM snapshots: cyan - cancer cells, grey – doomed cancer cells, with irreparable RT-induced damage, yellow - effector immune cells, red - regulatory immune cells).

| mIHC slide 12, parameter set 15, treated with $2Gy \times35$ WTRT | | |
| --- | --- | --- |
|  | 7 days prior to tumor clearance  (p-value) | 1 day prior to tumor clearance  (p-value) |
| Effector vs Tx | 3.85525E-06 | 1.62323E-07 |
| Effector vs Apoptosis | 1.52024E-07 | 3.46094E-08 |
| Tx vs Apoptosis | 3.60809E-08 | 0.242124283 |

**Table S7.** P-values of the two-sided Wilcoxon rank sum test, comparing the contribution of effector-mediated cancer cell death, treatment, and apoptosis in the 7 day or 1 day leading up to and including clearance of mIHC slide 12, parameter set 15 treated with 2Gy x 35 WTRT (see **Fig. 7**).

| mIHC slide 63, parameter set 13, treated with $2Gy \times35$ SFRT-GRID (30:70) | | |
| --- | --- | --- |
|  | 7 days prior to tumor clearance  (p-value) | 1 day prior to tumor clearance  (p-value) |
| Effector vs Tx | 7.0597E-18 | 5.43451E-18 |
| Effector vs Apoptosis | 7.05333E-18 | 6.79694E-18 |
| Tx vs Apoptosis | 0.404178298 | 0.005359835 |

**Table S8.** P-values of the two-sided Wilcoxon rank sum test, comparing the contribution of effector-mediated cancer cell death, treatment, and apoptosis in the 7 day or 1 day leading up to and including clearance of mIHC slide 63, parameter set 13 treated with 2Gy x 35 SFRT-GRID (30:70) (see **Fig. 8**).

| mIHC slide 63, parameter set 13, treated with $2Gy \times35$ SFRT-GRID (50:50) | | |
| --- | --- | --- |
|  | 7 days prior to tumor clearance  (p-value) | 1 day prior to tumor clearance  (p-value) |
| Effector vs Tx | 3.85525E-06 | 1.62323E-07 |
| Effector vs Apoptosis | 1.52024E-07 | 3.46094E-08 |
| Tx vs Apoptosis | 3.60809E-08 | 0.242124283 |

**Table S9.** P-values of the two-sided Wilcoxon rank sum test, comparing the contribution of effector-mediated cancer cell death, treatment, and apoptosis in the 7 day or 1 day leading up to and including clearance of mIHC slide 63, parameter set 13 treated with 2Gy x 35 SFRT-GRID (50:50) (see **Fig. 8**).


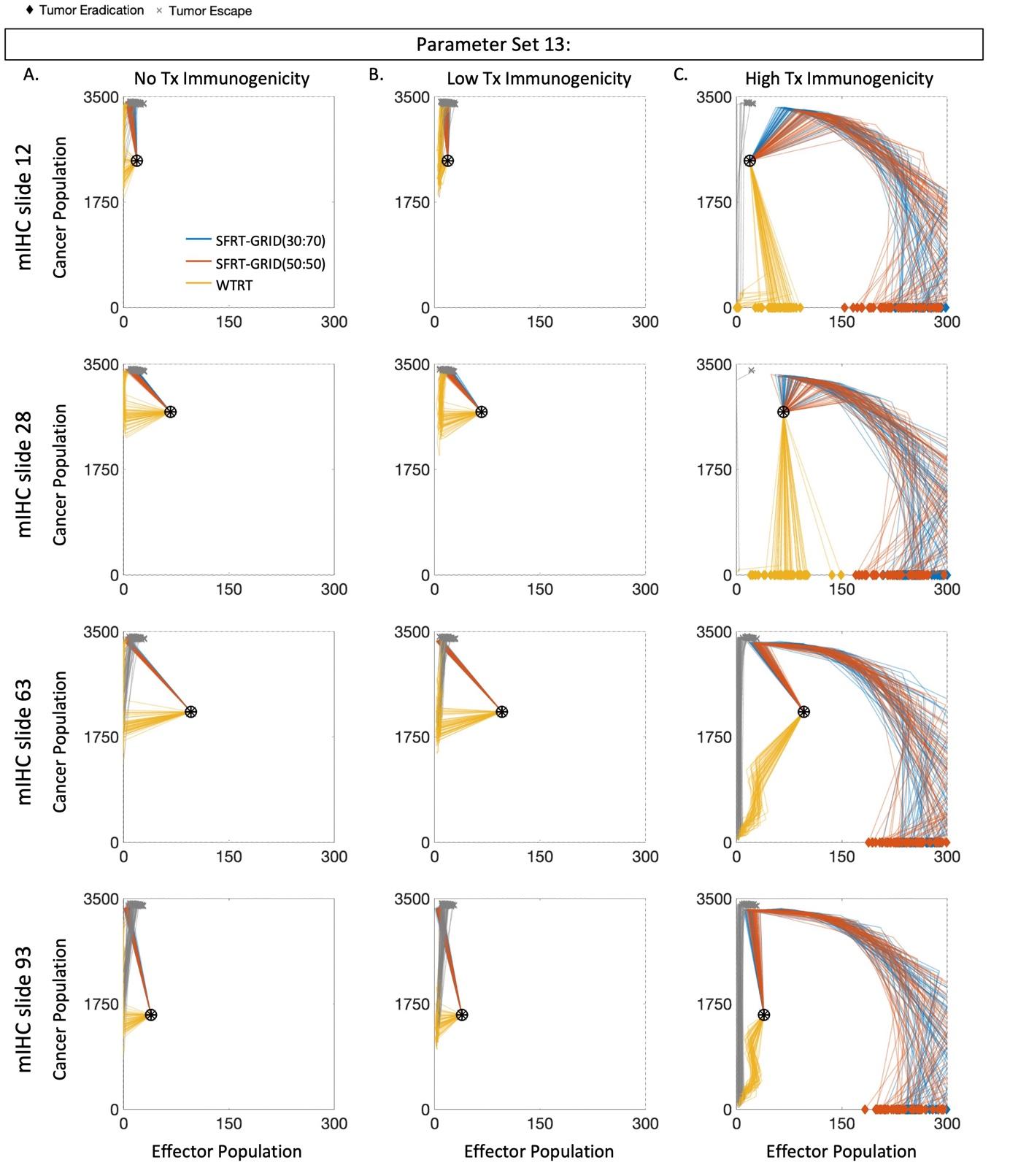


**Fig. S8 Cancer-Effector Planes of each mIHC slides during treatment with 2Gy x 35, for parameters set 13.**

1. Treatment is not immunogenic ${(\zeta}_{Tx}=0)$.
2. Treatment has low immunogenicity ${(\zeta}_{Tx}=0.01)$.
3. Treatment is highly immunogenic ${(\zeta}_{Tx}=0.1)$.


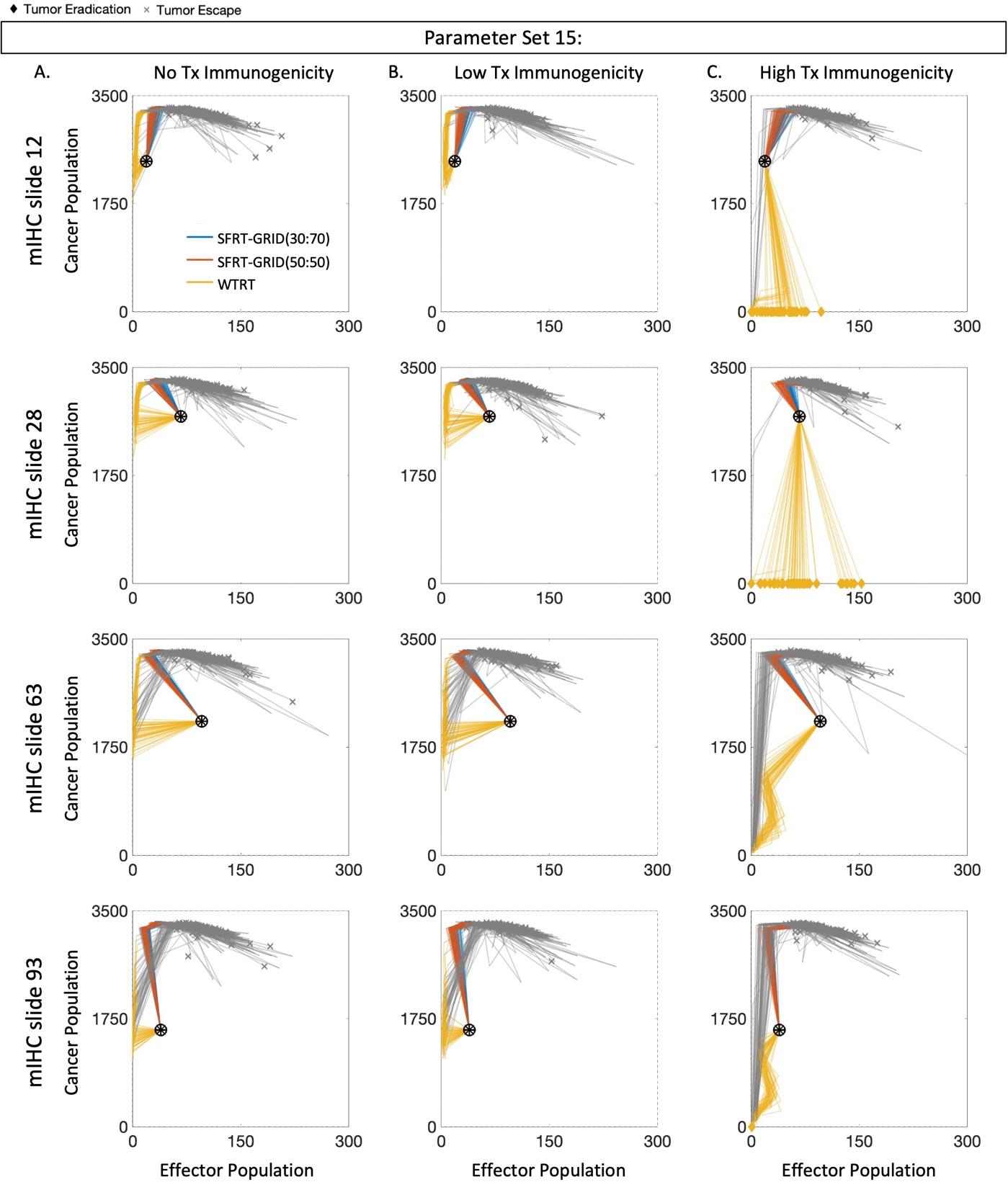


**Fig. S9 Cancer-Effector Planes of each mIHC slides during treatment with 2Gy x 35, for parameters set 15.**

1. Treatment is not immunogenic ${(\zeta}_{Tx}=0)$.
2. Treatment has low immunogenicity ${(\zeta}_{Tx}=0.01)$.
3. Treatment is highly immunogenic ${(\zeta}_{Tx}=0.1)$.

| Parameter set 13 | | | | |
| --- | --- | --- | --- | --- |
| mIHC Slide | SFRT-GRID (30:70) | SFRT-GRID (50:50) | Combo SFRT-GRID (30:70) | Combo SFRT-GRID (50:50) |
| 12 | 1 | 1 | 0.94 | 1 |
| 28 | 1 | 1 | 1 | 1 |
| 63 | 1 | 1 | 0.18 | 0.68 |
| 93 | 1 | 1 | 0 | 0.04 |
| Parameter set 15 | | | | |
| 12 | 0 | 0 | 1 | 1 |
| 28 | 0 | 0 | 1 | 1 |
| 63 | 0 | 0.02 | 0.08 | 0.90 |
| 93 | 0 | 0 | 0 | 0.04 |

**Table S10.** Treatment Efficacy of fractionated SFRT-GRID ($2Gy \times35$) vs clinical ablative SFRT-GRID ($15Gy \times1$ SFRT-GRID followed by $2Gy \times25$ WTRT), for parameter sets 13 and 15, for mIHC slides 12, 28, 63 and 93.

| mIHC slide 63, parameter set 13, treated with clinical ablative schedule:  $15Gy \times1 SFRT-GRID (50:50) followed by 2Gy \times25 WTRT.$ | | |
| --- | --- | --- |
|  | 7 days prior to tumor clearance (p-value) | 1 day prior to tumor  clearance (p-value) |
| Effector vs Tx | 8.77115E-05 | 0.297982874 |
| Effector vs Apoptosis | 0.00045415 | 0.001627988 |
| Tx vs Apoptosis | 3.9767E-06 | 6.63324E-08 |

**Table S11.** P-values of the two-sided Wilcoxon rank sum test, comparing the contribution of effector-mediated cancer cell death, treatment, and apoptosis in the 7 day or 1 day leading up to and including clearance of mIHC slide 63, parameter set 13 treated with the clinical ablative SFRT-GRID schedule. See Fig S10.


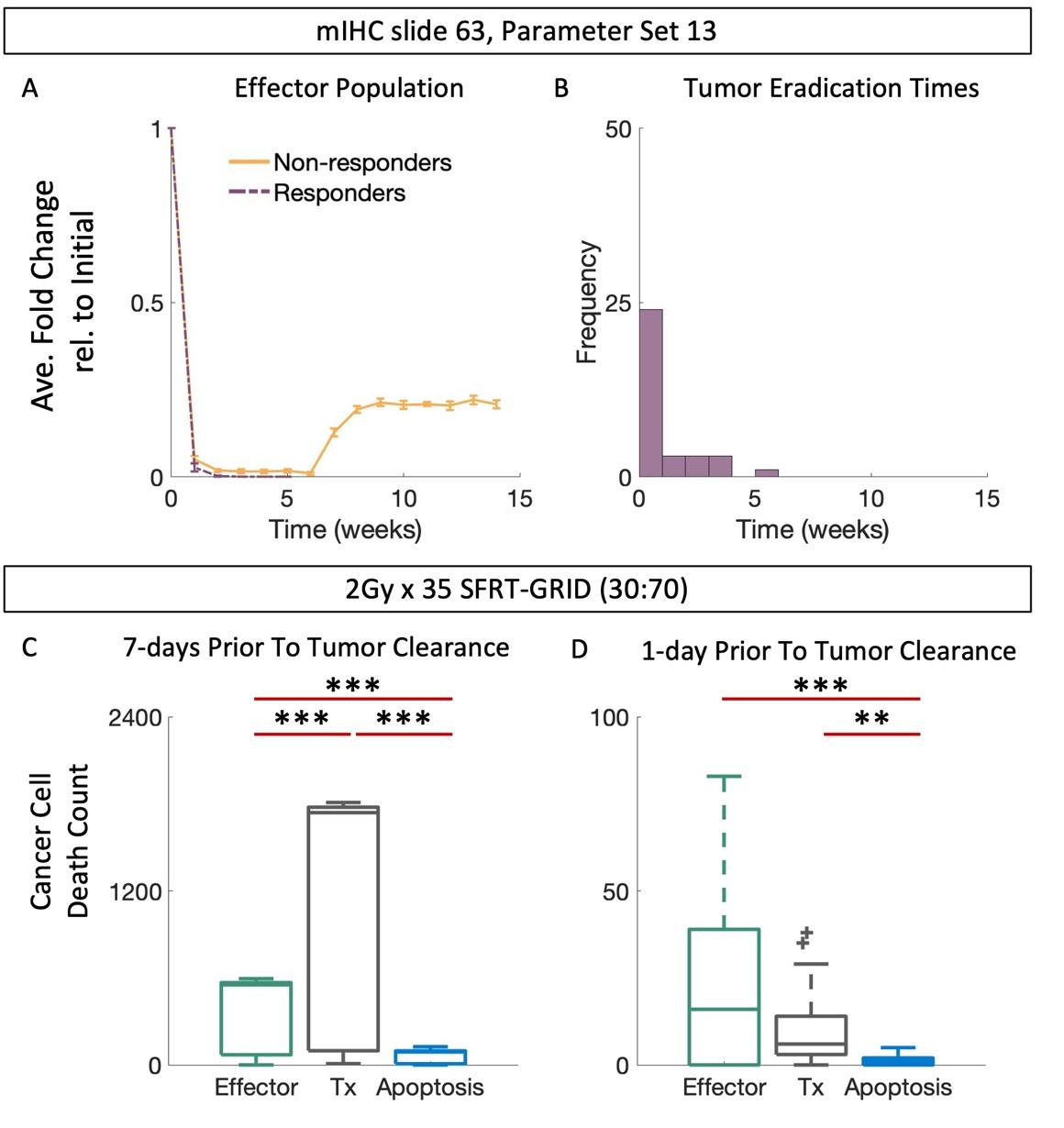


**Fig. S10 Clinical Ablative SFRT-GRID schedules debulk the tumor and suppress the immune effector population. A.** Average fold change of effector population in responding or non-responding mIHC slide 63 tumors treated with $15Gy \times1$SFRT-GRID (50:50) followed by $2Gy \times25$ WTRT. **B.** Distribution of clearance times for responding tumors in A. **C-D.** Contribution of effector-mediated cancer cell death, treatment, and apoptosis in the 7 day or 1 day period leading up to and including clearance of tumors treated with the clinical ablative SFRT-GRID schedule. Error bars are mean $\pm$ SEM. (* p<0.005, two-sided Wilcoxon rank sum test, Table S11).

**References:**

1. Chaplain MAJ, Graziano L, Preziosi L. Mathematical modelling of the loss of tissue compression responsiveness and its role in solid tumour development. Mathematical Medicine and Biology: A Journal of the IMA. 2006;23(3):197-229.

2. Enderling H, Anderson Ar Fau - Chaplain MAJ, Chaplain Ma Fau - Beheshti A, Beheshti A Fau - Hlatky L, Hlatky L Fau - Hahnfeldt P, Hahnfeldt P. Paradoxical dependencies of tumor dormancy and progression on basic cell kinetics. 2009(1538-7445 (Electronic)).

3. Enderling H, Hlatky L Fau - Hahnfeldt P, Hahnfeldt P. Migration rules: tumours are conglomerates of self-metastases. 2009(1532-1827 (Electronic)).

4. Weigelin B, den Boer AT, Wagena E, Broen K, Dolstra H, de Boer RJ, et al. Cytotoxic T cells are able to efficiently eliminate cancer cells by additive cytotoxicity. Nature Communications. 2021;12(1):5217.

5. Beck RJ, Bijker DI, Beltman JB. Heterogeneous, delayed-onset killing by multiple-hitting T cells: Stochastic simulations to assess methods for analysis of imaging data. PLoS Comput Biol. 2020;16(7):e1007972.

6. Singh K, Stempora L, Harvey RD, Kirk AD, Larsen CP, Blazar BR, et al. Superiority of rapamycin over tacrolimus in preserving nonhuman primate Treg half-life and phenotype after adoptive transfer. Am J Transplant. 2014;14(12):2691-703.

7. Miller MJ, Wei SH, Parker I, Cahalan MD. Two-Photon Imaging of Lymphocyte Motility and Antigen Response in Intact Lymph Node. Science. 2002;296(5574):1869-73.

8. Mueller SN. Effector T-cell responses in non-lymphoid tissues: insights from in vivo imaging. Immunology & Cell Biology. 2013;91(4):290-6.

9. Bougherara H, Mansuet-Lupo A, Alifano M, Ngô C, Damotte D, Le Frère-Belda M-A, et al. Real-Time Imaging of Resident T Cells in Human Lung and Ovarian Carcinomas Reveals How Different Tumor Microenvironments Control T Lymphocyte Migration. Frontiers in Immunology. 2015;6.

10. Alfonso JCL, Grass GD, Welsh E, Ahmed KA, Teer JK, Pilon-Thomas S, et al. Tumor-immune ecosystem dynamics define an individual Radiation Immune Score to predict pan-cancer radiocurability. Neoplasia (New York, NY). 2021;23(11):1110-22.
